# Associations between treatments, comorbidities and multidimensional aspects of quality of life among patients with advanced cancer in the Netherlands—a 2017–2020 multicentre cross-sectional study

**DOI:** 10.1007/s11136-023-03460-8

**Published:** 2023-06-30

**Authors:** Ananya Malhotra, Heidi P. Fransen, Manuela Quaresma, Natasja Raijmakers, Moyke A. J. Versluis, Bernard Rachet, Marissa C. van Maaren, Clémence Leyrat

**Affiliations:** 1 Inequalities in Cancer Outcomes Network, Department of Non-Communicable Disease Epidemiology, Faculty of Epidemiology and Population Health, London School of Hygiene & Tropical Medicine, London, UK; 2 Department of Research and Development, Netherlands Comprehensive Cancer Organisation (IKNL), Utrecht, The Netherlands; 3 Netherlands Association for Palliative Care (PZNL), Utrecht, The Netherlands; 4 Graduate School of Social & Behavioral Sciences, Universiteit Van Tilburg, Warandelaan 2, 5037 AB Tilburg, Nederland; 5 Department of Health Technology and Services Research, Technical Medical Centre, University of Twente, Enschede, The Netherlands; 6 Department of Medical Statistics, Faculty of Epidemiology and Population Health, London School of Hygiene & Tropical Medicine, London, UK

**Keywords:** Quality of life, Immunotherapy, Cancer treatments, Comorbidities, Advanced cancer

## Abstract

**Objective:**

To investigate associations between quality of life (QoL) and 1) immunotherapy and other cancer treatments received three months before QoL measurements, and 2) the comorbidities at the time of completion or in the year prior to QoL measurements, among patients with advanced cancer.

**Methods:**

A cross-sectional study is conducted on patients with advanced cancer in the Netherlands. The data come from the baseline wave of the 2017–2020 eQuiPe study. Participants were surveyed via questionnaires (including EORTC QLQ-C30). Using multivariable linear and logistic regression models, we explored statistical associations between QoL components and immunotherapy and other cancer treatments as well as pre-existing comorbidities while adjusting for age, sex, socio-economic status.

**Results:**

Of 1088 participants with median age 67 years, 51% were men. Immunotherapy was not associated with global QoL but was associated with reduced appetite loss (odds ratio (OR) = 0.6, 95%CI = [0.3,0.9]). Reduced global QoL was associated with chemotherapy (adjusted mean difference (β) = − 4.7, 95% CI [− 8.5,− 0.8]), back pain (β = − 7.4, 95% CI [− 11.0,− 3.8]), depression (β = − 13.8, 95% CI [− 21.5,− 6.2]), thyroid diseases (β = − 8.9, 95% CI [− 14.0,− 3.8]) and diabetes (β = − 4.5, 95% CI [− 8.9,− 0.5]). Chemotherapy was associated with lower physical (OR = 2.4, 95% CI [1.5,3.9]) and role (OR = 1.8, 95% CI [1.2,2.7]) functioning, and higher pain (OR = 1.9, 95% CI [1.3,2.9]) and fatigue (OR = 1.6, 95% CI [1.1,2.4]).

**Conclusion:**

Our study identified associations between specific cancer treatments, lower QoL and more symptoms. Monitoring symptoms may improve QoL of patients with advanced cancer. Producing more evidence from real life data would help physicians in better identifying patients who require additional supportive care.

**Supplementary Information:**

The online version contains supplementary material available at 10.1007/s11136-023-03460-8.

## Introduction

Despite significant advances in medical oncology, more than a fifth of patients with cancer are diagnosed with metastases in the Netherlands [1]. Such patients often have a poor prognosis and need palliative care. Palliative care has been defined by the WHO as ‘an approach that improves the quality of life (QoL) of patients and their families who are facing problems associated with life-threatening illness’, such as metastasized cancer.

In palliative care settings, it is thus imperative to evaluate the effects of cancer treatments not only on patient’s survival, but also on their QoL. Traditionally, the effectiveness of cancer treatments has been evaluated in terms of disease-free and overall survival, and changes in tumour characteristics. More recently, the assessment of patient-reported physical and psychosocial outcomes has gained considerable importance, and it is now incorporated within many clinical trials [2–5], leading to a direct impact on clinical practice [6].

A paradigm shift has been observed in palliative treatments with the introduction of immunotherapy, but the determinants of its benefits on quality of life, adverse effects and survival are scarcely explored. Some randomised clinical trials have shown that side-effects of immunotherapy are often better tolerated than those of traditional cytotoxic chemotherapy [7], but it remains unclear whether this can be translated into a better QoL.

Therefore, identifying determinants of QoL, including adverse effects, is key to maintain or improve patients’ health related QoL [8], and in turn reduce hospitalisation. QoL is partly determined by patient- and tumour-related factors such as their socio-demographic characteristics, comorbidities and cancer treatments, and partly by psychological determinants, including coping mechanisms [9]. Population-based studies have revealed that patients’ socio-demographic characteristics such as age, sex and marital status may impact their QoL [10–12]. This was also observed in a randomised trial among advanced cancer patients [13]. Hence, the effect of cancer treatments on QoL outcomes may be confounded by such factors. Very little has been systematically done regarding post-treatment QoL in relation with immunotherapy [14]. There is limited published evidence from observational real-world data focusing on the impact of immunotherapy as well as of other cancer treatments such as chemotherapy, radiotherapy an d surgery on QoL [10–12]. Moreover, patients with advanced cancer may have comorbidities which in turn have differential impact on their QoL [15]. The role of such comorbidities in the QoL of patients with advanced cancer patients has not been well studied. Using data from the eQuiPe study in the Netherlands, our aim is to investigate the associations between QoL components and 1) immunotherapy and other cancer treatments received three months before QoL measurements, and 2) the comorbidities at the time of completion or in the year prior to QoL measurements.

## Methods

### Data source

The data comes from the eQuiPe study [16] conducted by the Netherlands Comprehensive Cancer Organisation (IKNL) in the Netherlands. Our analysis is cross-sectional and based on the baseline data of the eQuiPe study, which is originally a prospective longitudinal observational cohort of patients with metastatic cancer aiming to understand their QoL and improve care. eQuiPe is a nationwide study conducted in forty hospitals all over the Netherlands, where eligible patients filled questionnaires every three months regarding their QoL during their care. These questionnaires were filled between November 2017 to March 2020 and were further linked to the Netherlands Cancer registry (NCR) which is hosted by IKNL and is a nationwide population-based registry including all malignancies diagnosed since 1989. Trained data managers register patient-, tumour- and treatment-related characteristics directly from patient files. Questionnaires were used to collect information on possible additional treatments that were administered after diagnosis of the metastasis.

### Inclusion criteria

Patients included in the eQuiPe study were aged 18 or over, able to complete a Dutch self-report questionnaire, understood the objectives of the eQuiPe research and consented to participate. Included patients were diagnosed with (progression of) a solid tumour with metastases (stage IV)[17] between 1988–2020. All sites of the primary tumour were considered for this study, including tumours of the respiratory and intrathoracic organs, digestive organs, male genital organs, and breast. However, additional criteria were applied for patients with breast or prostate cancer to minimise variation in life expectancy based on primary tumour type and overrepresentation of patients who have advanced cancer with relatively good prognosis. Hence, only breast cancer patients with metastases located in multiple organ systems and prostate cancer patients with metastasised and castration-resistant cancers were eligible. Patients who suffered from dementia or had a history of severe psychiatric illness were excluded.

### Questionnaire

The EORTC QLQ-C30[18] questionnaire (version 3) is an integrated system for assessing the health related QoL of cancer patients participating in international clinical trials. This 30-item questionnaire assesses all 15 QoL components, namely, the global QoL, 5 functional scales (emotional, physical, social, role and cognitive), 8 symptom scales (pain, fatigue, nausea/vomiting, dyspnoea, insomnia, loss of appetite, constipation and diarrhoea) and perceived financial impact of the disease. Some QoL components are derived from multiple questions, which are discussed in the EORTC QLQ-C30 scoring manual [18]. The responses are recorded as raw scores on the Likert scale (1 = not at all, 2 = a little, 3 = quite a bit, 4 = very much). Using linear transformations, these scores are converted to a continuous measure of scale ranging between 0 to 100 with higher scores representing higher global QoL/higher level of functioning/ higher level of symptoms.

Subsequently, socio-demographic variables, comorbidities, cancer treatments, recent hospitalisation and current symptoms were included in the eQuiPe questionnaire.

### Outcome and covariables

Our main outcome of interest is QoL of patients diagnosed with advanced cancer.

There are two groups of exposures of interest, the first one being recent cancer treatments in the last three months since the survey which includes immunotherapy, chemotherapy, radiotherapy, surgery and other treatments (like endocrine therapy). The second group of exposure is presence of individual comorbidities as listed in Table 1, either at the time of first questionnaire or developed in the past one year since the completion of the survey. Each of the treatments and comorbidities are coded separately as binary variables indicating presence/absence of treatment/comorbidity.

**Table 1:** Clinical and demographic characteristics of the study population out of 1,088 patients with advanced cancer at baseline

	Number of patients	Missing
	n (%)	n (%)
*Demographic characteristics*		
Sex		
Male	553 (50.8%)	0 (0%)
Age		
Mean (SD)	66.1 (9.8)	47 (4.3%)
Median [Q1, Q3]	67.0 [60.0, 73.0]	
Socio-economic status		
Low	297 (27.3%)	79 (7.3%)
Medium	438 (40.3%)	
High	274 (25.1%)	
^1^*Cancer treatments in the last 3 months*		
No treatment	423 (38.9%)	8 (0.7%)
Surgery	298 (27.4%)	7 (0.6%)
Immunotherapy	208 (19.1%)	8 (0.7%)
Chemotherapy	144 (13.2%)	7 (0.6%)
Other therapy^2^	58 (5.3%)	7 (0.6%)
Radiotherapy	37 (3.4%)	7 (0.6%)
*Comorbidities in the past 1 year*		
Heart condition	128 (11.8%)	39 (3.6%)
Stroke/CVA	17 (1.6%)	40 (3.7%)
High blood pressure	238 (21.9%)	39 (3.6%)
Asthma/Chronic bronchitis/COPD	108 (9.9%)	40 (3.7%)
Diabetes	107 (9.8%)	39 (3.6%)
Ulcer	11 (1.0%)	41 (3.8%)
Kidney Disease	31 (2.8%)	40 (3.7%)
Liver disease	28 (2.6%)	40 (3.7%)
Anaemia/Other blood condition	52 (4.8%)	40 (3.7%)
Thyroid disease	73 (6.7%)	39 (3.6%)
Depression	31 (2.8%)	40 (3.7%)
Arthritis	63 (5.8%)	40 (3.7%)
Back pain	170 (15.6%)	40 (3.7%)
Rheumatism	53 (4.9%)	41 (3.8%)
Other comorbidities	168 (15.4%)	30 (2.8%)
*Tumour site*		
Respiratory and intrathoracic organs	332 (30.5%)	
Digestive organs	308 (28.3%)	
Breast	168 (15.4%)	
Male genital organs	129 (11.9%)	
Other organs^3^	151 (13.9%)	

*CVA* cerebrovascular accident, *COPD* chronic obstructive pulmonary disease, *SD* standard deviation, *Q1* 1^st^ quartile, *Q3* 3^rd^ quartile. ^1^Some patients (83, 7.6%) received more than one type of treatments, hence the proportion receiving treatments in Table 1 do not add up to 1. Other treatments^2^: targeted therapy, hormonal therapy, stem cell transplantation or other unknown treatment. Other organs^3^: Female genital organs, Urinary tract, Skin, Mesothelial and soft tissue, Eye, brain, other parts of central nervous system, Lip, oral cavity and pharynx, Thyroid and other endocrine

Covariables included demographic features such as sex, age (years), socio-economic status (SES) and marital status at the time of completion of the survey (in a relationship or single/widowed). SES was based on scores assigned to the four numbers of the Dutch postal code, extracted from the Netherlands Institute for Social Research. The scores arise from a principal component analysis on mean household income, percentage of inhabitants with a low income, percentage of low educatedness and percentage of unemployment [19]. The score was consequently coded into deciles. For this analysis, a SES score of 1–3 was recoded as ‘low’, 4–7 was ‘medium’ and 8–10 was recoded as ‘high’ SES.

Since the NCR holds no record on subsequent treatments (after nine months from diagnosis) given at the time of a metachronous metastasis (only if it is present at primary diagnosis), we do not include the treatments recorded by NCR and restrict our analysis to treatments based on the survey responses only.

### Statistical analyses

Multivariable linear and logistic regression models were used to investigate the associations between 1) recent cancer treatments and different QoL components, and 2) comorbidities and QoL components, while controlling for possible confounder variables. A set of covariables sufficient for controlling the effects of confounding were selected via d-separation rules using a Directed Acyclic Graph (DAG). Although the limitations of the data do not allow us to draw causal conclusions, the DAGs (created using DAGitty v3.0 [20]) helped to identify important adjustment factors. For our analysis, we formed two separate DAGs, one to establish the adjusted total effects of recent cancer treatments on QoL components (Figure A.1) and the other to establish the effects of comorbidities on QoL components (Figure A.2). In both the DAGs, the exposure, outcome, other covariables and their associations known from the literature were first graphically depicted. Covariables – age, sex and SES were identified as adjustment factors in both the DAGs. Additionally, comorbidities confounded the effect of cancer treatments on QoL (Figure A.1), the recent cancer treatments were mediators in the path of comorbidities and QoL (Figure A.2) and hence were excluded from this analysis.

Model 1.1 depicts a multivariable linear regression model to study the effect of cancer treatments in the last three months on global QoL at baseline, adjusted for confounders like comorbidities in the past one year, age, sex and SES. Model 2.1 depicts a multivariable linear regression model to study the effect of comorbidities in the past one year on the global QoL at baseline while adjusting for age, sex and SES.

The scores on all 14 QoL components were not continuously distributed and hence, linear regression models are not an appropriate choice to model these outcomes. The functional scale scores are dichotomised as high/low level of functioning, symptom scale scores are dichotomised as presence/absence of symptom and financial difficulties score is dichotomised as yes/no, all using clinically relevant threshold values [21] (Table 2B,C). In Model 1.2, we fit 14 separate multivariable logistic regression models to study the effect of cancer treatments on the various QoL components, adjusted for comorbidities, age, sex and SES and in Model 2.2, we fit 14 separate multivariable logistic regression models to study the effect of comorbidities on the various QoL components. A histogram with a superimposed normal curve was used to check the normality of residuals assumption of linear models and the Hosmer–Lemeshow [22] test was used to assess goodness of fit of the logistic regression models. We did not perform multiple testing as our analysis was exploratory.

**Table 2:** Summary of quality of life scores of the study population of out of 1,088 participants

A		
Baseline QoL	Score*	Missing values n (%)
Global QoL score		
Mean (SD)	67.7 (20.0)	23 (2.1%)
Median [Q1, Q3]	66.7 [58.3,83.3]	

B			
Functional scale	Threshold score* of clinical importance of functioning	Number of patients, n (%) out of 1088	
		With low level of functioning	Missing information on functioning
Physical	< 83	679 (62.4%)	23 (2.1%)
Role	< 58	367 (33.7%)	26 (2.4%)
Emotional	< 71	340 (31.3%)	23 (2.1%)
Cognitive	< 75	325 (29.9%)	23 (2.1%)
Social	< 58	181 (16.6%)	24 (2.2%)

C			
Symptom scale	Threshold score* of clinical importance of symptom	Number of patients, n (%) out of 1088	
		With clinically relevant symptom	Missing information on symptom
Dyspnoea	≥ 17	509 (46.8%)	26 (2.4%)
Fatigue	≥ 39	450 (41.3%)	24 (2.2%)
Pain	≥ 25	401 (36.9%)	23 (2.1%)
Nausea/Vomiting	≥ 8	340 (31.3%)	23 (2.1%)
Diarrhoea	≥ 17	279 (24.6%)	29 (2.7%)
Insomnia	≥ 50	207 (19.0%)	26 (2.4%)
Loss of appetite	≥ 50	151 (13.9%)	26 (2.4%)
Constipation	≥ 50	79 (7.3%)	25 (2.3%)

D			
Financial impact	Threshold score* of clinical importance	Number of patients, n (%) out of 1088	
		With high financial impact	Missing information on financial impact
Yes	≥ 17	235 (21.6%)	23 (2.1%)

*Score out of 100, *QoL* quality of life, *SD* standard deviation, *Q1* 1st quartile, Q3: 3rd quartile

Since the variable `depression’ is a component of the emotional functioning outcome, we excluded it while estimating the effects of cancer treatments and comorbidities on the `emotional’ functioning. Similarly, comorbidities like back pain and asthma were excluded from the models with outcome variable ‘pain’ and `dyspnoea’ respectively.

Subgroup analyses by tumour site (Appendix B) were performed to assess potential differences in the effect of exposure on QoL. The ICD-10 codes of primary tumours were categorised by site and only the top two most prevalent tumour sites were considered for subgroup analysis.

All analyses were performed using RStudio software version 4.0.4 [23].

## Results

The population comprises 1,089 patients diagnosed with solid metastasised (stage IV) primary tumours of any site, between 1988–2020. Patients’ characteristics, tumour site, recent cancer treatments in the previous three months and comorbidities in the past year are presented in Table 1. One participant did not meet the criteria of diagnosis of solid primary tumour and was thus excluded leaving us with 1,088 participants. Nearly equal number of men (n = 554,51%) and women with median age of 67 years [IQR = (60,73)] at baseline participated. There were 657 (60.4%) patients who had treatment(s) in the last three months prior to the baseline survey, 298 (27.4%) underwent surgery, a fifth received immunotherapy (n = 208,19.1%), 144 (13.2%) received chemotherapy, 58 (5.3%) received other treatments and 37 (3.4%) received radiotherapy. Comorbidities such as high blood pressure (238,21.9%) and back pain (170,15.6%) were most prevalent. Table 2 presents the results for the global QoL scores (mean (SD) and median [IQR]) and dichotomised scores of functional and symptom scales using clinical thresholds [21]. The median global QoL score was 66.7 [58.3,83.3].

### Association between treatment and QoL

**Fig. 1:**
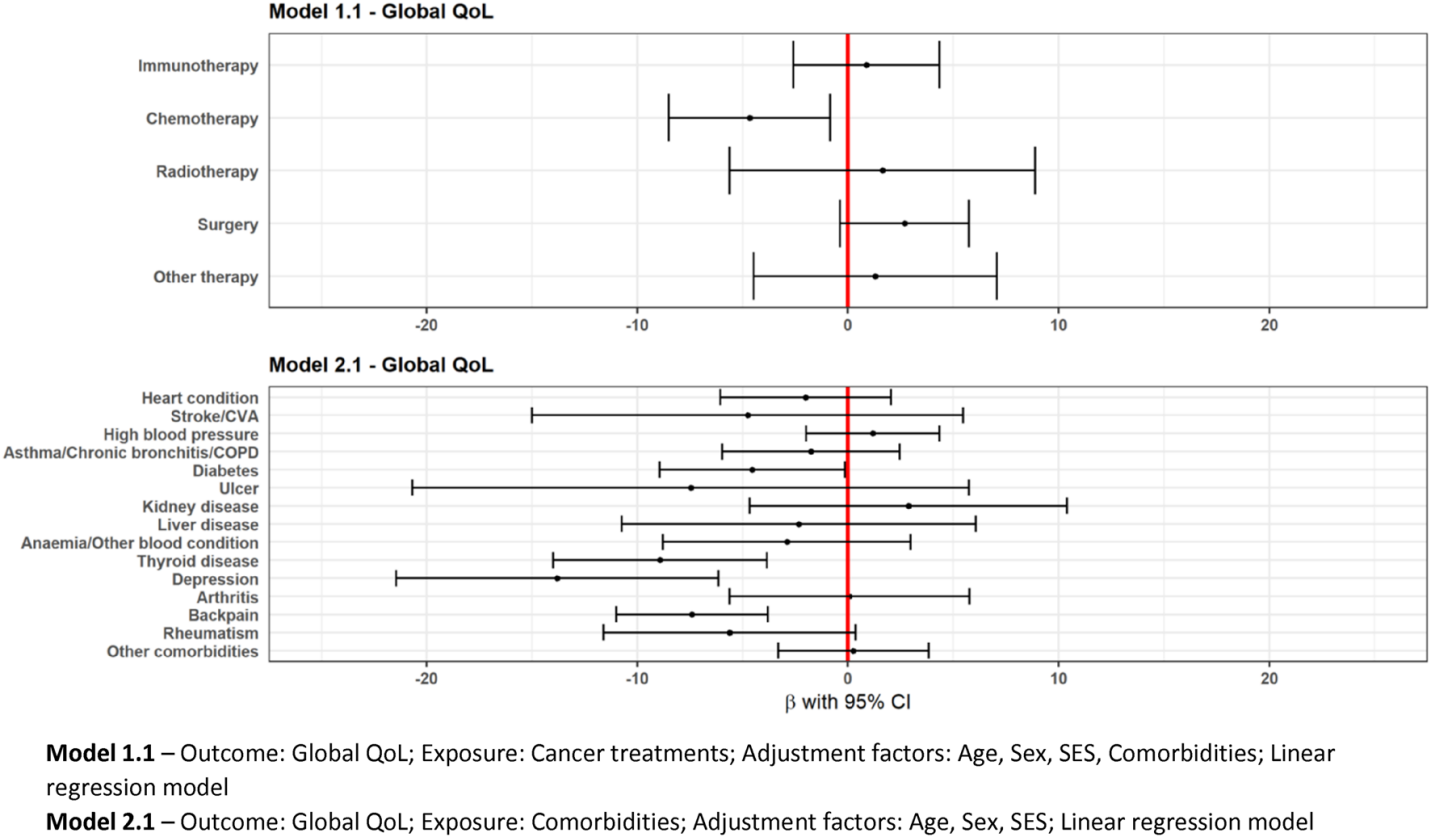
Forest plot of regression coefficients (β) from multivariable linear regression showing the adjusted differences in mean global QoL scores and their 95% confidence intervals (CI)

**Fig. 2:**
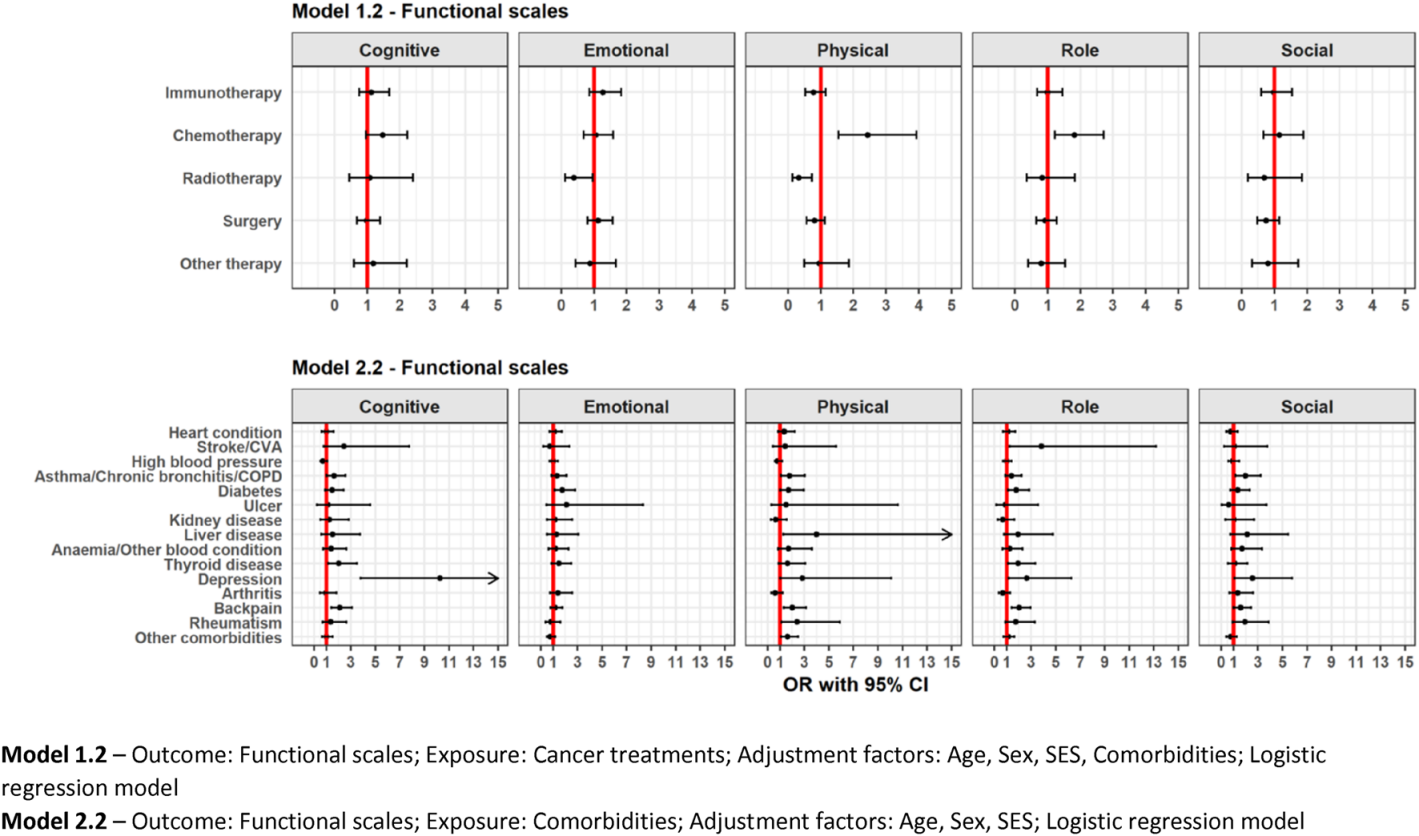
Forest plot of odds ratios (OR) from multivariable logistic regression on the presence of clinically relevant problems on functional scale scores and their 95% CI

**Fig. 3:**
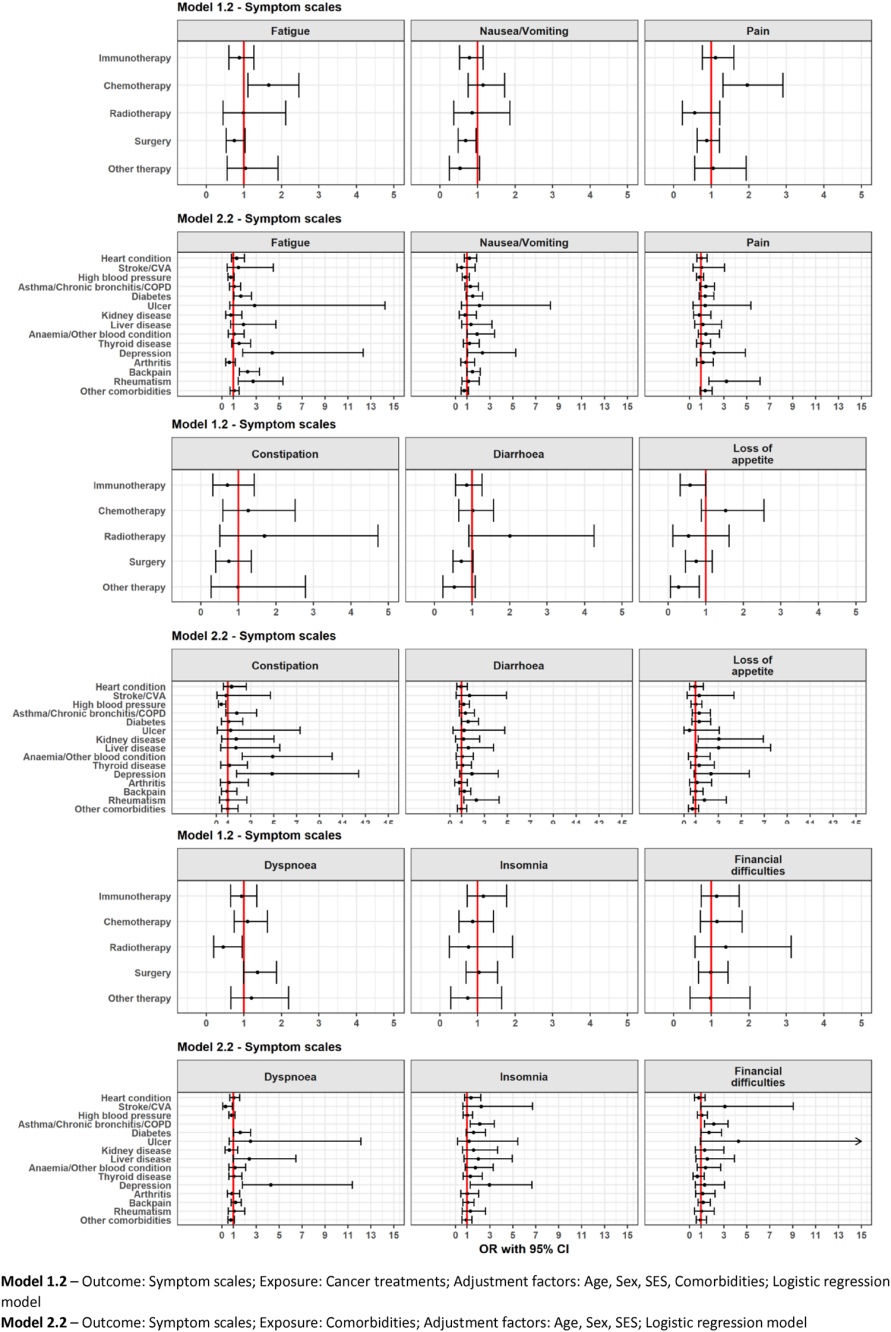
Forest plot of OR from multivariable logistic regression on the presence of clinically relevant symptom scale scores and their 95% CI

Treatments are differently associated with global QoL. Immunotherapy treatment (vs no immunotherapy) was not associated with a lower or higher global QoL (Model 1.1, Fig. 1). No clear association was found between immunotherapy and having clinically relevant problems on the different functional components (Model 1.2, Fig. 2), while immunotherapy had lower odds of clinically relevant appetite loss (OR = 0.6,95%CI = [0.3,0.9]) (Fig. 3). Recent chemotherapy was associated with a lower global QoL (β = − 4.7,95%CI = [− 8.5,− 0.8]) and also related to higher odds of low role functioning (OR = 1.8,95% CI = [1.2,2.7]) and low physical functioning (OR = 2.4,95%CI = [1.5,3.9]). Moreover, chemotherapy was related with higher odds of more fatigue (OR = 1.5,95%CI = [1.1,2.4]) and more pain symptoms (OR = 1.9,95%CI = [1.3,2.9]). Radiotherapy was related to lower odds of low emotional (OR = 0.4,95%CI = [0.1,0.9]) and low physical functioning (OR = 0.3,95%CI = [0.1,0.7]) and more dyspnoea (OR = 0.4,95%CI = [0.2,0.9]).

The results from subgroup analysis overall generally agreed with the full analysis. Among patients with respiratory and intrathoracic tumours, chemotherapy was associated with reduced social functioning and increased nausea/vomiting symptoms while radiotherapy was associated with better global QoL (Table B.1). Among patients with cancers of digestive organs, there was evidence of an association between radiotherapy and increased diarrhoea symptoms while immunotherapy was related to lower diarrhoea (Table B.2).

### Association between comorbidities and QoL

Analysis of comorbidities revealed a strong association between the presence of comorbidities and global QoL in patients with advanced cancer (Model 2.1), such as back pain (β = − 7.4,95%CI = [− 11,− 3.8]), depression (β = − 13.8,95%CI = [− 21.4,− 6.1]), thyroid disease (β = − 8.9,95%CI = [− 14,− 3.8]) and diabetes (β = − 4.5,95%CI = [− 8.9,− 0.1]). Back pain and depression showed a strong association with physical, role and cognitive functioning (Model 2.2, Fig. 2). Asthma/COPD was strongly associated with higher odds of low social (OR = 1.9,95%CI = [1.2,3.2]), low physical (OR = 1.8,95%CI = [1.1,3]) and low cognitive (OR = 1.6,95%CI = [1,2.6]) functioning as well as more insomnia (OR = 2,95%CI = [1.2,3.4]). Anaemia/other blood conditions were associated with higher odds of more constipation (OR = 4.9,95%CI = [2.2,10]). There was no association between global QoL and comorbidities like arthritis, ulcers and stroke/CVA. The full results are shown in Figs. 1, 2 and 3.

All linear models satisfied the normality of residuals assumptions and all logistic regression models were a good fit as per the Hosmer–Lemeshow goodness of fit test.

## Discussion

Patient-reported outcomes and QoL indicators are paramount in assessing the impact of treatments and comorbidities on the QoL of cancer patients. This cross-sectional study was primarily aimed at finding associations between immunotherapy and other cancer treatments and QoL of patients with advanced cancer. Such patients are restricted in their QoL due to their diagnosis and potential treatment-related side-effects. Physicians are aware of this fact [24] and research efforts as well as clinical practice are being mounted towards optimising patients’ QoL. Identification of cancer treatments and comorbidities associated with QoL measures may help minimise the decline in patients’ QoL.

Our study showed that over a third of patients scored below the threshold level on the physical and role functioning scales and above the threshold level on dyspnoea, fatigue and pain symptom scales. Moreover, studies with more focused populations with respect to cancer sites also reported symptoms including fatigue [25], dyspnea [25], pain [25] and insomnia [25, 26] after treatment for advanced cancer. We also observed associations between specific treatments and QoL components, as also observed in the limited literature, for example among breast cancer survivors [15].

QoL of cancer patients with advanced cancer following immunotherapy has only received limited and recent attention in research, despite its importance [27, 28]. Our statistical analysis did not find strong associations between immunotherapy and lower global QoL or its functional components. However, immunotherapy maybe associated with less appetite loss, although there was weak evidence for it. To the best of our knowledge, there are no studies indicating lower appetite loss after immunotherapy while loss of appetite has been commonly reported after chemotherapy, radiotherapy or surgery [29]. Moreover, PD-(L)1 inhibitors like nivolumab, pembrolizumab and atezolizumab have been observed to be associated with consistent delay in time to symptomatic deterioration in QoL among patients with solid tumours [30] compared to traditional cytotoxic therapy. This was also reflected in our study where we could not see a significant impact of immunotherapy on QoL at baseline while chemotherapy was associated with poorer global QoL, and lower physical and role functioning. This association has also been observed among patients with breast or colon cancer [31] and colorectal cancer [32] who were assessed before and after their first cycle of chemotherapy. However, we did not observe any significant association between chemotherapy and emotional, social and cognitive functioning in this study. Pain is a common side-effect of chemotherapy [33, 34] which was also observed in our study. In contrast with a single-centre study in Brazil with 84% women with breast cancer [35], our study did not show significant positive association between chemotherapy and constipation symptoms. Another study on rectal cancer patients showed that radiotherapy significantly increased diarrhoea, fatigue and appetite loss and reduced physical and role functioning and global QoL at the end of radiotherapy [36]. In our data, we observed that radiotherapy was associated with increased diarrhoea among a broader category of patients with cancer of the digestive organs, possibly reflecting radiation enteritis of which diarrhoea is one of the most commonly reported symptoms (Table B.2). It is also expected to observe increased nausea and vomiting in patients with respiratory and intrathoracic tumours who received chemotherapy. In our data, lung cancer represented 97% of these tumours, for which the most common chemotherapy drug combinations used include cisplatin, and often carboplatin, which are both emetogenic.

Our analysis did not indicate any significant association between immunotherapy and lower QoL during or just after administration as opposed to chemotherapy. Early integration with palliative care has shown better patient outcomes in terms of improved care through frequent monitoring of symptoms and functional status, timely intervention for troubling symptoms, better QoL and prolonged survival [37]. Evidence from clinical trials suggests that immunotherapy is generally well-tolerated but this evidence is mostly based on patients selected on their good performance status [38]. It can also be attributed to the fact that immunotherapy maybe administered to patients who are in better health condition.

Higher levels of social support given to patients is associated with better QoL [39], and social support tends to decrease with lower SES [40], which highlights the importance of the role of social support in more deprived patients. Moreover, effective social support systems can help reduce feelings of social isolation so patients can strive for normality [41].

### Strengths and limitations

In addition to the reasonable large study population, this nation-wide collaborative study also included a range of teaching and general hospitals and a good geographic spread, likely to be representative of the various care settings in the Netherlands.

The recruitment of patients in this study was completed in the year 2020 before the Covid-19 pandemic, however, the follow-up of patients was affected. In this study we have analysed the baseline data, which is not affected by the pandemic. The cross-sectional design of this study allowed us only to estimate associations with QoL, instead of any causal relationships. There is scope for selection bias as patients with better outcomes/ higher QoL were more likely to participate in the study than patients with lower QoL [42]. Moreover, this selection bias may have increased if the health care professional asked only patients with higher QoL to take part in the study. If the condition of the patient deteriorated after treatment, their participation would have been dramatically reduced. The length of the questionnaire could have led to a higher drop-out rate, especially among patients with more symptoms or poorer QoL. However, a meta-analysis showed no obvious indication that response rates are attributable to the length of the questionnaire [43]. This analysis is based on patient-reported cancer treatments, and it is possible that some patients may not be fully aware of their treatments. Moreover, we lacked information on the complete treatment history and only considered cancer treatments administered in the last three months, which might have affected our results. Lastly, some patients may have expressed difficulty in quantifying their level of symptoms [44].

### Conclusions and recommendations for clinical practice

Our findings suggest that immunotherapy was not associated with lower QoL, while chemotherapy was associated with lower QoL. As chemotherapy was associated with more pain and fatigue, monitoring these symptoms is important and may improve QoL of patients with advanced cancer. The associations of comorbidities such as back pain, diabetes, thyroid diseases and depression with lower QoL might also help identify patients who require additional care. In addition, it is vital to discuss both the advantages and potential harms of palliative treatments on QoL with patients in the treatment decision-making process and those should be re-evaluated regularly. However, decision making is largely informed by randomised trial data, which are heavily biased due to their strict inclusion criteria. It implies that evaluation of expected benefits and harms (e.g. by measuring QoL) based on real life data is urgently needed. The impact of social support on QoL can be further explored with the eQuiPe study.

### Supplementary Information

Below is the link to the electronic supplementary material.

Supplementary file1 (DOCX 1905 KB)

Supplementary file2 (DOCX 107 KB)

## Data Availability

Since 2011, PROFILES registry data is freely available according to the FAIR (Findable, Accessible, Interoperable, Reusable) data principles for non-commercial (international) scientific research, subject only to privacy and confidentiality restrictions. The datasets analysed during the current study are available through Questacy (DDI 3.x XML) and can be accessed by our website (www.profilesregistry.nl). In order to arrange optimal long-term data warehousing and dissemination, we follow the quality guidelines that are formulated in the ‘Data Seal of Approval’ (www.datasealofapproval.org) document, developed by Data Archiving and Networked Services (DANS). The underlying data of this manuscript would be made available when the eQuiPe study is completed.
