# Supplementary figures and images for: Associations between treatments, comorbidities and multidimensional aspects of quality of life among patients with advanced cancer in the Netherlands—a 2017–2020 multicentre cross-sectional study
Source: Qual Life Res. 2023 Jun 30;32(11):3123–33. doi: 10.1007/s11136-023-03460-8 (PMC10522740; doi:10.1007/s11136-023-03460-8)

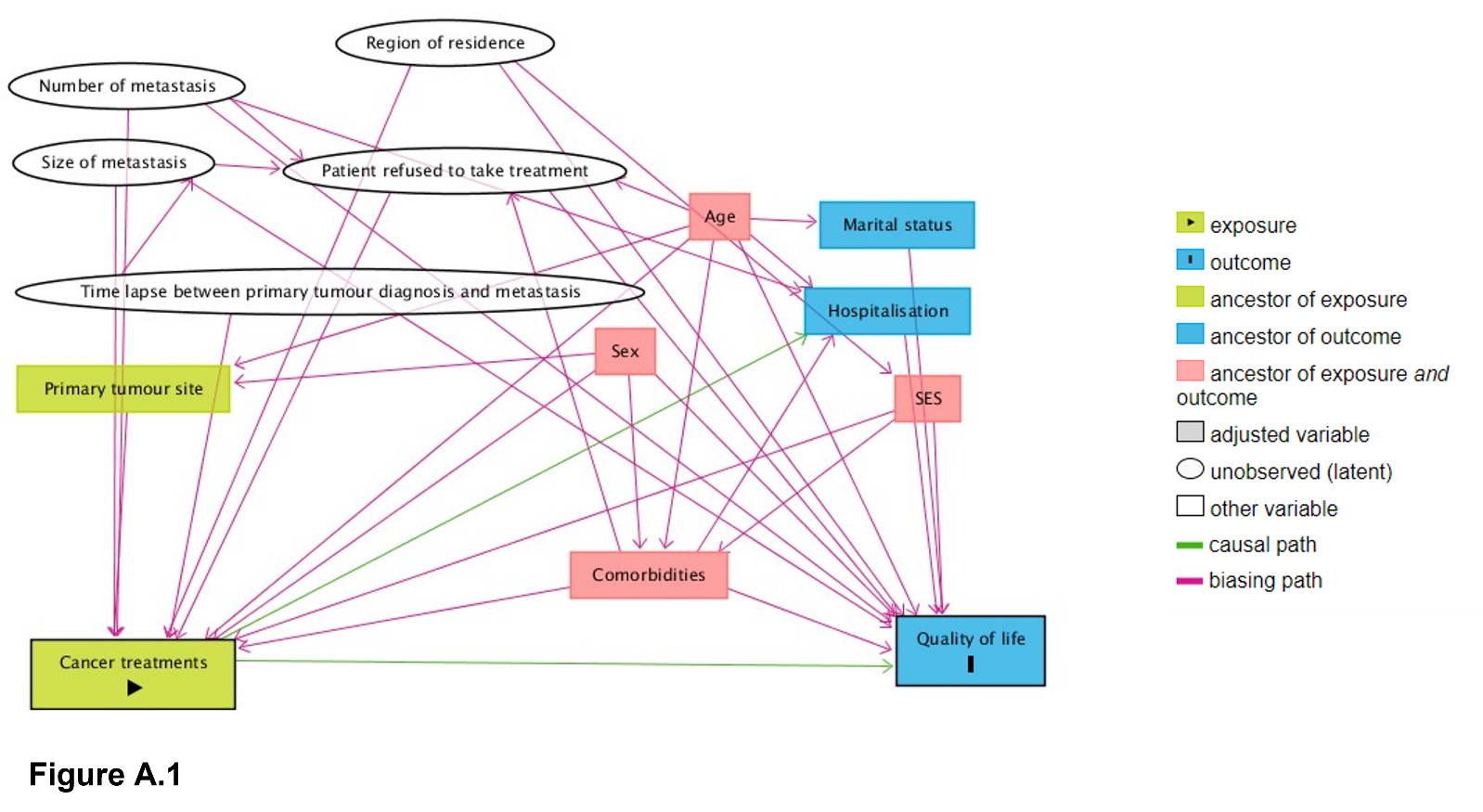


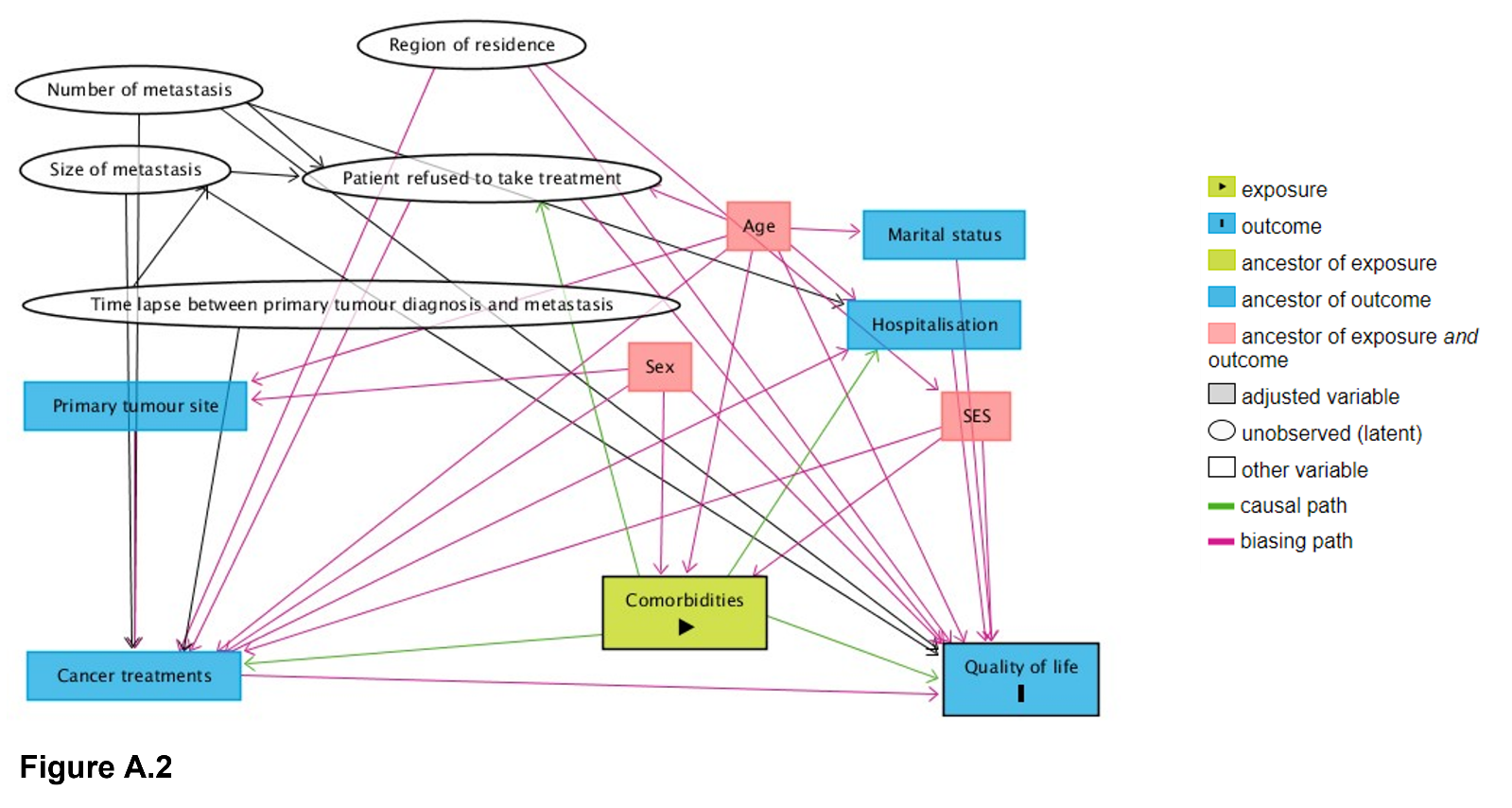

Supplement: Supplementary file 1 — Supplementary file1 (DOCX 1905 KB) [file 11136_2023_3460_MOESM1_ESM.docx]
