# Supplementary material for: Associations between treatments, comorbidities and multidimensional aspects of quality of life among patients with advanced cancer in the Netherlands—a 2017–2020 multicentre cross-sectional study
Source: Qual Life Res. 2023 Jun 30;32(11):3123–33. doi: 10.1007/s11136-023-03460-8 (PMC10522740; doi:10.1007/s11136-023-03460-8)
Supplement: Supplementary file 2 — Supplementary file2 (DOCX 107 KB) [file 11136_2023_3460_MOESM2_ESM.docx]

**Subgroup analyses by tumour site**

Among all tumour sites in this study, the most common were respiratory and intrathoracic cancers (n=332, 30.5%) of which 97.3% were lung and bronchus cancers and the remaining were thymus, heart, mediastinum and pleural cancers. The second most prevalent were cancers of the digestive organs (n=308, 28.3%) of which 39.6% were colon, 21.4% were rectum, 12.6% were oesophagus, 8% were pancreas, 6.8% were stomach and the remaining were rectosigmoid junction, small intestine, liver, gallbladder and biliary duct. Other tumour sites included breast cancers (168, 15.4%), cancers of the male (129, 11.8%) and female (53, 4.8%) genital organs, urinary tract (41, 3.7%), skin (21, 2%) and other cancers.

As observed among all cancer sites, a subgroup analysis among patients with respiratory and intrathoracic tumours also showed no evidence of an association between immunotherapy and global QoL but an association between chemotherapy and global QoL (β=-9.8, 95%CI=[-16.1,-3.5]) was significant. There was no evidence of an association between immunotherapy and reduced appetite loss among this subgroup of cancers (OR=0.5, 95%CI=[0.1,1.7])as opposed to all cancers. Chemotherapy was related to decrease in role (OR=2.4, 95%CI=[1.2,4.7]) and physical (OR=2.4, 95%CI=[1.1,5.4]) functioning. However, among this sub-group of patients, as opposed to all cancers, chemotherapy was also associated with reduced social functioning (OR=4.1, 95%CI=[1.7,9.9]) and increased nausea/vomiting (OR=2.2, 95%CI=[1.1,4.4]) symptoms while radiotherapy was associated with better global QoL (β=17.8, 95%CI=[0.7,34.9]). As in all cancers, thyroid and depression were associated with global QoL but not diabetes and back pain. The full results are shown in Table B.1.

**Table B.1 - Tumours of the respiratory and intrathoracic organs**

| **Model 1.1** |  |  | **95% CI** | |  |
| --- | --- | --- | --- | --- | --- |
| **Outcome** | **Exposure** | **β** | **Lower** | **Upper** | **p-value** |
| Overall QoL | Intercept | 85.880 | 65.122 | 106.638 | <0.001 |
| Overall QoL | Immunotherapy | 7.491 | -0.413 | 15.395 | 0.063 |
| Overall QoL | Chemotherapy | -9.790 | -16.106 | -3.474 | 0.003 |
| Overall QoL | Radiotherapy | 17.850 | 0.728 | 34.973 | 0.041 |
| Overall QoL | Surgery | 3.858 | -1.288 | 9.005 | 0.141 |
| Overall QoL | Other therapy | 1.384 | -11.590 | 14.357 | 0.834 |
| **Model 1.2** |  |  | **95% CI** | |  |
| **Outcome** | **Exposure** | **OR** | **Lower** | **Upper** | **p-value** |
| Emotional | Intercept | 1.072 | 0.104 | 10.629 | 0.953 |
| Emotional | Immunotherapy | 1.245 | 0.615 | 2.475 | 0.535 |
| Emotional | Chemotherapy | 0.239 | 0.011 | 1.915 | 0.236 |
| Emotional | Radiotherapy | 1.247 | 0.696 | 2.252 | 0.460 |
| Emotional | Surgery | 1.138 | 0.456 | 2.711 | 0.774 |
| Emotional | Other therapy | 0.303 | 0.015 | 1.918 | 0.285 |
| Social | Intercept | 0.021 | 0.001 | 0.604 | 0.031 |
| Social | Immunotherapy | 0.587 | 0.123 | 2.083 | 0.448 |
| Social | Chemotherapy | 4.105 | 1.715 | 9.924 | 0.001 |
| Social | Radiotherapy | - | - | - | - |
| Social | Surgery | 0.495 | 0.212 | 1.120 | 0.096 |
| Social | Other therapy | 0.668 | 0.032 | 4.764 | 0.729 |
| Role | Intercept | 0.496 | 0.044 | 5.246 | 0.564 |
| Role | Immunotherapy | 0.600 | 0.230 | 1.460 | 0.274 |
| Role | Chemotherapy | 2.379 | 1.203 | 4.731 | 0.013 |
| Role | Radiotherapy | 0.147 | 0.006 | 1.227 | 0.126 |
| Role | Surgery | 0.605 | 0.335 | 1.081 | 0.091 |
| Role | Other therapy | 0.328 | 0.044 | 1.527 | 0.196 |
| Physical | Intercept | 0.012 | 0.001 | 0.148 | 0.001 |
| Physical | Immunotherapy | 0.929 | 0.376 | 2.376 | 0.874 |
| Physical | Chemotherapy | 2.396 | 1.127 | 5.406 | 0.028 |
| Physical | Radiotherapy | 0.143 | 0.013 | 1.169 | 0.081 |
| Physical | Surgery | 0.643 | 0.346 | 1.179 | 0.157 |
| Physical | Other therapy | 0.657 | 0.160 | 2.776 | 0.557 |
| Cognitive | Intercept | 0.609 | 0.049 | 7.028 | 0.694 |
| Cognitive | Immunotherapy | 0.749 | 0.273 | 1.885 | 0.553 |
| Cognitive | Chemotherapy | 1.213 | 0.573 | 2.500 | 0.605 |
| Cognitive | Radiotherapy | 1.052 | 0.122 | 6.764 | 0.959 |
| Cognitive | Surgery | 0.653 | 0.348 | 1.212 | 0.179 |
| Cognitive | Other therapy | 0.903 | 0.189 | 3.769 | 0.891 |
| Fatigue | Intercept | 0.196 | 0.018 | 1.928 | 0.169 |
| Fatigue | Immunotherapy | 0.529 | 0.207 | 1.262 | 0.163 |
| Fatigue | Chemotherapy | 1.559 | 0.793 | 3.059 | 0.195 |
| Fatigue | Radiotherapy | 0.760 | 0.086 | 5.037 | 0.783 |
| Fatigue | Surgery | 0.700 | 0.397 | 1.226 | 0.214 |
| Fatigue | Other therapy | 0.817 | 0.188 | 3.161 | 0.775 |
| Pain | Intercept | 0.638 | 0.055 | 6.926 | 0.715 |
| Pain | Immunotherapy | 0.415 | 0.125 | 1.158 | 0.115 |
| Pain | Chemotherapy | 1.951 | 0.961 | 3.932 | 0.062 |
| Pain | Radiotherapy | 0.919 | 0.113 | 5.682 | 0.930 |
| Pain | Surgery | 1.205 | 0.666 | 2.193 | 0.539 |
| Pain | Other therapy | 1.847 | 0.443 | 7.377 | 0.383 |
| Nausea/Vomiting | Intercept | 0.629 | 0.057 | 6.512 | 0.700 |
| Nausea/Vomiting | Immunotherapy | 0.461 | 0.156 | 1.189 | 0.129 |
| Nausea/Vomiting | Chemotherapy | 2.240 | 1.131 | 4.425 | 0.020 |
| Nausea/Vomiting | Radiotherapy | 0.490 | 0.054 | 3.157 | 0.474 |
| Nausea/Vomiting | Surgery | 0.730 | 0.406 | 1.308 | 0.291 |
| Nausea/Vomiting | Other therapy | 1.470 | 0.324 | 6.056 | 0.598 |
| Dyspnoea | Intercept | 0.239 | 0.025 | 2.227 | 0.211 |
| Dyspnoea | Immunotherapy | 1.080 | 0.468 | 2.566 | 0.859 |
| Dyspnoea | Chemotherapy | 0.967 | 0.499 | 1.901 | 0.920 |
| Dyspnoea | Radiotherapy | 0.645 | 0.096 | 4.161 | 0.635 |
| Dyspnoea | Surgery | 0.955 | 0.546 | 1.667 | 0.872 |
| Dyspnoea | Other therapy | 4.432 | 0.935 | 33.704 | 0.090 |
| Insomnia | Intercept | 0.400 | 0.022 | 6.427 | 0.525 |
| Insomnia | Immunotherapy | 0.647 | 0.172 | 1.949 | 0.472 |
| Insomnia | Chemotherapy | 0.826 | 0.320 | 1.942 | 0.675 |
| Insomnia | Radiotherapy | - | - | - | - |
| Insomnia | Surgery | 0.869 | 0.428 | 1.758 | 0.695 |
| Insomnia | Other therapy | 0.632 | 0.032 | 4.185 | 0.685 |
| Loss of appetite | Intercept | 0.066 | 0.002 | 1.373 | 0.090 |
| Loss of appetite | Immunotherapy | 0.535 | 0.118 | 1.759 | 0.350 |
| Loss of appetite | Chemotherapy | 1.917 | 0.810 | 4.365 | 0.127 |
| Loss of appetite | Radiotherapy | - | - | - | - |
| Loss of appetite | Surgery | 0.627 | 0.295 | 1.312 | 0.217 |
| Loss of appetite | Other therapy | 0.286 | 0.013 | 2.013 | 0.286 |
| Constipation | Intercept | 0.209 | 0.004 | 7.791 | 0.417 |
| Constipation | Immunotherapy | 0.422 | 0.022 | 2.424 | 0.425 |
| Constipation | Chemotherapy | 1.351 | 0.396 | 4.013 | 0.604 |
| Constipation | Radiotherapy | 1.184 | 0.045 | 11.793 | 0.899 |
| Constipation | Surgery | 1.151 | 0.433 | 3.106 | 0.778 |
| Constipation | Other therapy | 2.590 | 0.311 | 15.418 | 0.320 |
| Diarrhoea | Intercept | 2.879 | 0.200 | 40.340 | 0.431 |
| Diarrhoea | Immunotherapy | 0.776 | 0.257 | 2.070 | 0.628 |
| Diarrhoea | Chemotherapy | 0.997 | 0.424 | 2.209 | 0.995 |
| Diarrhoea | Radiotherapy | 0.499 | 0.023 | 3.879 | 0.559 |
| Diarrhoea | Surgery | 0.661 | 0.336 | 1.288 | 0.226 |
| Diarrhoea | Other therapy | 0.851 | 0.115 | 4.120 | 0.853 |
| Financial difficulties | Intercept | 2.330 | 0.179 | 30.003 | 0.515 |
| Financial difficulties | Immunotherapy | 1.264 | 0.464 | 3.227 | 0.632 |
| Financial difficulties | Chemotherapy | 2.136 | 1.019 | 4.418 | 0.042 |
| Financial difficulties | Radiotherapy | 1.866 | 0.280 | 12.848 | 0.507 |
| Financial difficulties | Surgery | 0.792 | 0.411 | 1.522 | 0.484 |
| Financial difficulties | Other therapy | 0.786 | 0.105 | 3.811 | 0.783 |
| **Model 2.1** |  |  | **95% CI** | |  |
| **Outcome** | **Exposure** | **β** | **Lower** | **Upper** | **p-value** |
| Overall QoL | Intercept | 84.903 | 64.532 | 105.275 | <0.001 |
| Overall QoL | Heart condition | -2.019 | -8.906 | 4.869 | 0.564 |
| Overall QoL | Stroke/CVA | -6.949 | -27.871 | 13.973 | 0.514 |
| Overall QoL | High blood pressure | -0.679 | -6.588 | 5.230 | 0.821 |
| Overall QoL | Asthma/Chronic bronchitis/COPD | -4.599 | -11.127 | 1.929 | 0.167 |
| Overall QoL | Diabetes | -5.157 | -13.478 | 3.164 | 0.223 |
| Overall QoL | Ulcer | -9.838 | -34.011 | 14.336 | 0.424 |
| Overall QoL | Kidney disease | 2.425 | -14.424 | 19.274 | 0.777 |
| Overall QoL | Liver disease | 1.592 | -28.263 | 31.447 | 0.916 |
| Overall QoL | Anaemia/Other blood condition | -5.214 | -20.092 | 9.664 | 0.491 |
| Overall QoL | Thyroid disease | -14.006 | -22.999 | -5.013 | 0.002 |
| Overall QoL | Depression | -20.742 | -35.904 | -5.581 | 0.008 |
| Overall QoL | Arthritis | -3.899 | -13.532 | 5.735 | 0.426 |
| Overall QoL | Backpain | -1.832 | -8.650 | 4.986 | 0.597 |
| Overall QoL | Rheumatism | -6.420 | -17.915 | 5.075 | 0.273 |
| Overall QoL | Other comorbidities | 0.418 | -6.727 | 7.563 | 0.908 |
| **Model 2.2** |  |  | **95% CI** | |  |
| **Outcome** | **Exposure** | **OR** | **Lower** | **Upper** | **p-value** |
| Emotional | Intercept | 0.906 | 0.099 | 8.040 | 0.930 |
| Emotional | Heart condition | 1.552 | 0.719 | 3.278 | 0.253 |
| Emotional | Stroke/CVA | 3.785 | 0.395 | 36.447 | 0.221 |
| Emotional | High blood pressure | 0.712 | 0.352 | 1.383 | 0.327 |
| Emotional | Asthma/Chronic bronchitis/COPD | 1.975 | 0.973 | 3.981 | 0.057 |
| Emotional | Diabetes | 0.717 | 0.267 | 1.783 | 0.488 |
| Emotional | Ulcer | - | - | - | - |
| Emotional | Kidney disease | 1.254 | 0.158 | 7.166 | 0.806 |
| Emotional | Liver disease | 2.109 | 0.067 | 64.776 | 0.633 |
| Emotional | Anaemia/Other blood condition | 1.923 | 0.343 | 9.801 | 0.428 |
| Emotional | Thyroid disease | 1.696 | 0.628 | 4.458 | 0.285 |
| Emotional | Arthritis | 1.392 | 0.470 | 3.860 | 0.532 |
| Emotional | Backpain | 0.758 | 0.332 | 1.627 | 0.491 |
| Emotional | Rheumatism | 0.387 | 0.076 | 1.453 | 0.193 |
| Emotional | Other comorbidities | 0.318 | 0.111 | 0.776 | 0.019 |
| Social | Intercept | 0.058 | 0.002 | 1.186 | 0.074 |
| Social | Heart condition | 0.700 | 0.193 | 2.058 | 0.547 |
| Social | Stroke/CVA | 2.025 | 0.085 | 21.641 | 0.586 |
| Social | High blood pressure | 0.634 | 0.233 | 1.539 | 0.340 |
| Social | Asthma/Chronic bronchitis/COPD | 2.969 | 1.296 | 6.663 | 0.009 |
| Social | Diabetes | 1.560 | 0.454 | 4.697 | 0.449 |
| Social | Ulcer | 2.497 | 0.097 | 36.548 | 0.507 |
| Social | Kidney disease | 1.745 | 0.082 | 13.130 | 0.637 |
| Social | Liver disease | 1.809 | 0.038 | 71.835 | 0.739 |
| Social | Anaemia/Other blood condition | 0.627 | 0.026 | 4.961 | 0.710 |
| Social | Thyroid disease | 1.906 | 0.544 | 5.832 | 0.278 |
| Social | Depression | 5.279 | 0.934 | 28.518 | 0.050 |
| Social | Arthritis | 1.028 | 0.212 | 3.640 | 0.969 |
| Social | Backpain | 1.170 | 0.435 | 2.856 | 0.742 |
| Social | Rheumatism | 0.604 | 0.079 | 2.802 | 0.565 |
| Social | Other comorbidities | 0.645 | 0.183 | 1.834 | 0.449 |
| Role | Intercept | 0.512 | 0.053 | 4.621 | 0.555 |
| Role | Heart condition | 0.979 | 0.462 | 2.019 | 0.954 |
| Role | Stroke/CVA | - | - | - | - |
| Role | High blood pressure | 1.133 | 0.592 | 2.127 | 0.702 |
| Role | Asthma/Chronic bronchitis/COPD | 1.145 | 0.560 | 2.292 | 0.704 |
| Role | Diabetes | 2.528 | 1.052 | 6.191 | 0.039 |
| Role | Ulcer | - | - | - | - |
| Role | Kidney disease | 0.382 | 0.018 | 2.736 | 0.410 |
| Role | Liver disease | 0.559 | 0.011 | 22.762 | 0.749 |
| Role | Anaemia/Other blood condition | 1.693 | 0.301 | 8.602 | 0.522 |
| Role | Thyroid disease | 2.426 | 0.933 | 6.477 | 0.070 |
| Role | Depression | 2.699 | 0.570 | 14.744 | 0.215 |
| Role | Arthritis | 0.880 | 0.298 | 2.427 | 0.808 |
| Role | Backpain | 1.816 | 0.879 | 3.739 | 0.104 |
| Role | Rheumatism | 2.086 | 0.588 | 7.355 | 0.244 |
| Role | Other comorbidities | 1.184 | 0.549 | 2.484 | 0.659 |
| Physical | Intercept | 0.017 | 0.001 | 0.172 | 0.001 |
| Physical | Heart condition | 0.758 | 0.352 | 1.671 | 0.482 |
| Physical | Stroke/CVA | - | - | - | - |
| Physical | High blood pressure | 0.617 | 0.316 | 1.205 | 0.155 |
| Physical | Asthma/Chronic bronchitis/COPD | 1.714 | 0.792 | 3.975 | 0.187 |
| Physical | Diabetes | 2.089 | 0.771 | 6.469 | 0.169 |
| Physical | Ulcer | - | - | - | - |
| Physical | Kidney disease | 0.525 | 0.077 | 3.491 | 0.492 |
| Physical | Liver disease | - | - | - | - |
| Physical | Anaemia/Other blood condition | 1.450 | 0.285 | 10.656 | 0.672 |
| Physical | Thyroid disease | 1.786 | 0.612 | 6.047 | 0.313 |
| Physical | Depression | - | - | - | - |
| Physical | Arthritis | 0.355 | 0.117 | 1.081 | 0.065 |
| Physical | Backpain | 1.417 | 0.635 | 3.377 | 0.409 |
| Physical | Rheumatism | 1.507 | 0.384 | 7.750 | 0.581 |
| Physical | Other comorbidities | 0.833 | 0.369 | 1.937 | 0.663 |
| Cognitive | Intercept | 0.526 | 0.046 | 5.501 | 0.596 |
| Cognitive | Heart condition | 1.671 | 0.763 | 3.593 | 0.191 |
| Cognitive | Stroke/CVA | 15.009 | 1.528 | 341.930 | 0.032 |
| Cognitive | High blood pressure | 0.739 | 0.356 | 1.471 | 0.402 |
| Cognitive | Asthma/Chronic bronchitis/COPD | 1.616 | 0.756 | 3.379 | 0.207 |
| Cognitive | Diabetes | 0.662 | 0.219 | 1.782 | 0.435 |
| Cognitive | Ulcer | 3.800 | 0.297 | 95.384 | 0.320 |
| Cognitive | Kidney disease | 4.039 | 0.660 | 24.573 | 0.115 |
| Cognitive | Liver disease | - | - | - | - |
| Cognitive | Anaemia/Other blood condition | 1.616 | 0.282 | 7.536 | 0.553 |
| Cognitive | Thyroid disease | 2.860 | 1.116 | 7.478 | 0.029 |
| Cognitive | Depression | 15.699 | 2.252 | 323.804 | 0.017 |
| Cognitive | Arthritis | 0.852 | 0.249 | 2.547 | 0.783 |
| Cognitive | Backpain | 2.923 | 1.397 | 6.163 | 0.004 |
| Cognitive | Rheumatism | 0.392 | 0.073 | 1.607 | 0.227 |
| Cognitive | Other comorbidities | 0.974 | 0.411 | 2.185 | 0.951 |
| Fatigue | Intercept | 0.185 | 0.019 | 1.638 | 0.135 |
| Fatigue | Heart condition | 1.626 | 0.796 | 3.342 | 0.182 |
| Fatigue | Stroke/CVA | 3.336 | 0.327 | 79.696 | 0.350 |
| Fatigue | High blood pressure | 0.711 | 0.370 | 1.330 | 0.294 |
| Fatigue | Asthma/Chronic bronchitis/COPD | 1.187 | 0.589 | 2.365 | 0.628 |
| Fatigue | Diabetes | 2.754 | 1.153 | 6.841 | 0.024 |
| Fatigue | Ulcer | - | - | - | - |
| Fatigue | Kidney disease | 1.018 | 0.130 | 5.812 | 0.985 |
| Fatigue | Liver disease | 0.263 | 0.002 | 16.807 | 0.558 |
| Fatigue | Anaemia/Other blood condition | 0.489 | 0.062 | 2.604 | 0.434 |
| Fatigue | Thyroid disease | 1.267 | 0.482 | 3.290 | 0.625 |
| Fatigue | Depression | 10.401 | 1.581 | 219.255 | 0.040 |
| Fatigue | Arthritis | 0.771 | 0.262 | 2.149 | 0.624 |
| Fatigue | Backpain | 1.716 | 0.838 | 3.539 | 0.140 |
| Fatigue | Rheumatism | 2.084 | 0.608 | 7.619 | 0.245 |
| Fatigue | Other comorbidities | 1.205 | 0.558 | 2.557 | 0.629 |
| Pain | Intercept | 0.915 | 0.090 | 8.825 | 0.939 |
| Pain | Heart condition | 1.205 | 0.550 | 2.559 | 0.633 |
| Pain | Stroke/CVA | 2.240 | 0.217 | 22.766 | 0.473 |
| Pain | High blood pressure | 0.531 | 0.253 | 1.057 | 0.081 |
| Pain | Asthma/Chronic bronchitis/COPD | 1.206 | 0.575 | 2.459 | 0.611 |
| Pain | Diabetes | 1.679 | 0.656 | 4.132 | 0.265 |
| Pain | Ulcer | 4.834 | 0.294 | 164.958 | 0.297 |
| Pain | Kidney disease | 0.792 | 0.040 | 5.422 | 0.837 |
| Pain | Liver disease | - | - | - | - |
| Pain | Anaemia/Other blood condition | 0.670 | 0.085 | 3.385 | 0.654 |
| Pain | Thyroid disease | 1.304 | 0.483 | 3.378 | 0.589 |
| Pain | Depression | 1.493 | 0.282 | 8.539 | 0.634 |
| Pain | Arthritis | 1.364 | 0.445 | 3.877 | 0.569 |
| Pain | Rheumatism | 3.820 | 1.156 | 13.793 | 0.031 |
| Pain | Other comorbidities | 3.107 | 1.471 | 6.615 | 0.003 |
| Nausea/Vomiting | Intercept | 0.698 | 0.074 | 6.272 | 0.749 |
| Nausea/Vomiting | Heart condition | 1.136 | 0.526 | 2.371 | 0.738 |
| Nausea/Vomiting | Stroke/CVA | 0.700 | 0.032 | 6.451 | 0.772 |
| Nausea/Vomiting | High blood pressure | 0.630 | 0.309 | 1.226 | 0.186 |
| Nausea/Vomiting | Asthma/Chronic bronchitis/COPD | 1.285 | 0.628 | 2.567 | 0.484 |
| Nausea/Vomiting | Diabetes | 1.578 | 0.640 | 3.797 | 0.311 |
| Nausea/Vomiting | Ulcer | - | - | - | - |
| Nausea/Vomiting | Kidney disease | 0.907 | 0.103 | 5.457 | 0.920 |
| Nausea/Vomiting | Liver disease | 1.131 | 0.028 | 39.901 | 0.942 |
| Nausea/Vomiting | Anaemia/Other blood condition | 3.441 | 0.691 | 18.868 | 0.128 |
| Nausea/Vomiting | Thyroid disease | 0.705 | 0.229 | 1.919 | 0.513 |
| Nausea/Vomiting | Depression | 1.663 | 0.324 | 8.248 | 0.525 |
| Nausea/Vomiting | Arthritis | 1.082 | 0.372 | 2.935 | 0.880 |
| Nausea/Vomiting | Backpain | 1.584 | 0.763 | 3.226 | 0.208 |
| Nausea/Vomiting | Rheumatism | 1.703 | 0.498 | 5.697 | 0.382 |
| Nausea/Vomiting | Other comorbidities | 0.635 | 0.261 | 1.417 | 0.289 |
| Dyspnoea | Intercept | 0.233 | 0.027 | 1.956 | 0.182 |
| Dyspnoea | Heart condition | 0.707 | 0.344 | 1.474 | 0.348 |
| Dyspnoea | Stroke/CVA | 0.115 | 0.005 | 1.210 | 0.095 |
| Dyspnoea | High blood pressure | 0.886 | 0.474 | 1.676 | 0.706 |
| Dyspnoea | Diabetes | 2.416 | 0.940 | 7.129 | 0.083 |
| Dyspnoea | Ulcer | 1.357 | 0.113 | 31.838 | 0.814 |
| Dyspnoea | Kidney disease | 0.226 | 0.028 | 1.297 | 0.110 |
| Dyspnoea | Liver disease | - | - | - | - |
| Dyspnoea | Anaemia/Other blood condition | 0.969 | 0.211 | 5.197 | 0.968 |
| Dyspnoea | Thyroid disease | 0.679 | 0.267 | 1.748 | 0.415 |
| Dyspnoea | Depression | 5.946 | 0.892 | 119.382 | 0.116 |
| Dyspnoea | Arthritis | 0.689 | 0.246 | 2.004 | 0.480 |
| Dyspnoea | Backpain | 1.288 | 0.625 | 2.762 | 0.502 |
| Dyspnoea | Rheumatism | 0.433 | 0.124 | 1.436 | 0.173 |
| Dyspnoea | Other comorbidities | 0.783 | 0.366 | 1.705 | 0.529 |
| Insomnia | Intercept | 0.261 | 0.016 | 3.704 | 0.332 |
| Insomnia | Heart condition | 2.074 | 0.857 | 4.813 | 0.095 |
| Insomnia | Stroke/CVA | 5.674 | 0.562 | 60.430 | 0.124 |
| Insomnia | High blood pressure | 1.586 | 0.735 | 3.306 | 0.226 |
| Insomnia | Asthma/Chronic bronchitis/COPD | 1.961 | 0.867 | 4.280 | 0.096 |
| Insomnia | Diabetes | 1.152 | 0.387 | 3.062 | 0.786 |
| Insomnia | Ulcer | 2.631 | 0.080 | 45.030 | 0.521 |
| Insomnia | Kidney disease | 0.933 | 0.046 | 6.700 | 0.952 |
| Insomnia | Liver disease | - | - | - | - |
| Insomnia | Anaemia/Other blood condition | 1.209 | 0.130 | 6.718 | 0.843 |
| Insomnia | Thyroid disease | 1.000 | 0.257 | 3.162 | 0.999 |
| Insomnia | Depression | 8.547 | 1.545 | 52.146 | 0.013 |
| Insomnia | Arthritis | 1.110 | 0.282 | 3.520 | 0.868 |
| Insomnia | Backpain | 0.992 | 0.369 | 2.398 | 0.986 |
| Insomnia | Rheumatism | 0.450 | 0.053 | 2.242 | 0.389 |
| Insomnia | Other comorbidities | 0.472 | 0.131 | 1.345 | 0.199 |
| Loss of appetite | Intercept | 0.069 | 0.003 | 1.178 | 0.074 |
| Loss of appetite | Heart condition | 1.157 | 0.413 | 2.911 | 0.767 |
| Loss of appetite | Stroke/CVA | 7.088 | 0.684 | 76.950 | 0.087 |
| Loss of appetite | High blood pressure | 0.591 | 0.223 | 1.387 | 0.253 |
| Loss of appetite | Asthma/Chronic bronchitis/COPD | 1.375 | 0.555 | 3.163 | 0.469 |
| Loss of appetite | Diabetes | 0.974 | 0.264 | 2.964 | 0.965 |
| Loss of appetite | Ulcer | 2.885 | 0.107 | 40.904 | 0.446 |
| Loss of appetite | Kidney disease | 5.370 | 0.859 | 32.994 | 0.060 |
| Loss of appetite | Liver disease | - | - | - | - |
| Loss of appetite | Anaemia/Other blood condition | 2.269 | 0.370 | 11.481 | 0.335 |
| Loss of appetite | Thyroid disease | 0.723 | 0.151 | 2.490 | 0.641 |
| Loss of appetite | Depression | 0.600 | 0.028 | 4.492 | 0.668 |
| Loss of appetite | Arthritis | 2.251 | 0.640 | 6.902 | 0.173 |
| Loss of appetite | Backpain | 0.984 | 0.359 | 2.396 | 0.974 |
| Loss of appetite | Rheumatism | 0.650 | 0.086 | 2.967 | 0.620 |
| Loss of appetite | Other comorbidities | 1.908 | 0.726 | 4.616 | 0.166 |
| Constipation | Intercept | 0.293 | 0.007 | 8.705 | 0.495 |
| Constipation | Heart condition | 1.469 | 0.365 | 4.837 | 0.551 |
| Constipation | Stroke/CVA | 11.450 | 0.426 | 172.869 | 0.085 |
| Constipation | High blood pressure | 0.351 | 0.064 | 1.256 | 0.154 |
| Constipation | Asthma/Chronic bronchitis/COPD | 0.591 | 0.111 | 2.135 | 0.473 |
| Constipation | Diabetes | 0.621 | 0.071 | 3.042 | 0.606 |
| Constipation | Ulcer | 8.994 | 0.272 | 180.338 | 0.154 |
| Constipation | Kidney disease | 1.524 | 0.063 | 13.020 | 0.737 |
| Constipation | Liver disease | - | - | - | - |
| Constipation | Anaemia/Other blood condition | 6.989 | 1.091 | 40.153 | 0.029 |
| Constipation | Thyroid disease | 0.356 | 0.018 | 2.119 | 0.354 |
| Constipation | Depression | 1.587 | 0.072 | 12.653 | 0.702 |
| Constipation | Arthritis | 1.230 | 0.161 | 5.724 | 0.813 |
| Constipation | Backpain | 1.333 | 0.343 | 4.153 | 0.644 |
| Constipation | Rheumatism | 0.437 | 0.019 | 3.218 | 0.497 |
| Constipation | Other comorbidities | 1.420 | 0.359 | 4.509 | 0.579 |
| Diarrhoea | Intercept | 1.799 | 0.139 | 22.204 | 0.648 |
| Diarrhoea | Heart condition | 0.683 | 0.239 | 1.719 | 0.443 |
| Diarrhoea | Stroke/CVA | 2.349 | 0.239 | 23.061 | 0.437 |
| Diarrhoea | High blood pressure | 1.767 | 0.858 | 3.554 | 0.115 |
| Diarrhoea | Asthma/Chronic bronchitis/COPD | 1.752 | 0.782 | 3.791 | 0.161 |
| Diarrhoea | Diabetes | 1.766 | 0.652 | 4.506 | 0.244 |
| Diarrhoea | Ulcer | 5.786 | 0.422 | 149.609 | 0.200 |
| Diarrhoea | Kidney disease | 0.848 | 0.041 | 6.370 | 0.888 |
| Diarrhoea | Liver disease | 1.981 | 0.065 | 61.067 | 0.659 |
| Diarrhoea | Anaemia/Other blood condition | 0.881 | 0.091 | 4.951 | 0.896 |
| Diarrhoea | Thyroid disease | 0.956 | 0.269 | 2.861 | 0.939 |
| Diarrhoea | Depression | 0.654 | 0.073 | 3.665 | 0.657 |
| Diarrhoea | Arthritis | 1.772 | 0.550 | 5.204 | 0.311 |
| Diarrhoea | Backpain | 0.774 | 0.301 | 1.801 | 0.570 |
| Diarrhoea | Rheumatism | 4.452 | 1.281 | 15.486 | 0.017 |
| Diarrhoea | Other comorbidities | 0.931 | 0.339 | 2.269 | 0.881 |
| Financial difficulties | Intercept | 4.351 | 0.387 | 50.469 | 0.234 |
| Financial difficulties | Heart condition | 0.387 | 0.121 | 1.015 | 0.074 |
| Financial difficulties | Stroke/CVA | 4.065 | 0.423 | 39.564 | 0.199 |
| Financial difficulties | High blood pressure | 1.019 | 0.486 | 2.052 | 0.958 |
| Financial difficulties | Asthma/Chronic bronchitis/COPD | 1.835 | 0.854 | 3.849 | 0.112 |
| Financial difficulties | Diabetes | 1.122 | 0.386 | 2.942 | 0.822 |
| Financial difficulties | Ulcer | 6.039 | 0.447 | 155.217 | 0.186 |
| Financial difficulties | Kidney disease | 1.307 | 0.167 | 7.446 | 0.771 |
| Financial difficulties | Liver disease | - | - | - | - |
| Financial difficulties | Anaemia/Other blood condition | 2.228 | 0.355 | 11.290 | 0.347 |
| Financial difficulties | Thyroid disease | 1.234 | 0.391 | 3.460 | 0.701 |
| Financial difficulties | Depression | 0.382 | 0.018 | 2.826 | 0.416 |
| Financial difficulties | Arthritis | 0.927 | 0.242 | 2.876 | 0.903 |
| Financial difficulties | Backpain | 1.014 | 0.427 | 2.248 | 0.973 |
| Financial difficulties | Rheumatism | 0.882 | 0.165 | 3.460 | 0.868 |
| Financial difficulties | Other comorbidities | 0.631 | 0.220 | 1.556 | 0.348 |
| “-“: very large point estimates (>100) with wide confidence interval of length > 200 | | | | | |

Among patients with cancers of the digestive organs, immunotherapy was not associated with global QoL as in all cancers but as opposed to all cancers, it was no longer associated with appetite loss. Chemotherapy was only associated with role functioning (OR=2.7, 95%CI=[1,7.3]). As opposed to all cancers, in this sub-group there was evidence of an association between radiotherapy and increased diarrhoea symptoms (OR=3.4, 95%CI=[1.1,11.4]) while immunotherapy was related to lower diarrhoea (OR=0.2, 95%CI=[0.02,0.8]) at baseline. Back pain and depression were still associated with global QoL in this sub-group but not thyroid and diabetes. The full results are shown in Table B.2.

There were no differences in these cancer specific populations with respect to associations between depression and reduced global QoL. Similarly, no differences in these populations were observed with regards to associations between back pain and reduced cognitive functioning, anaemia and increased constipation.

**Table B.2 – Tumours of the digestive organs**

| **Model 1.1** |  |  | **95% CI** | |  |
| --- | --- | --- | --- | --- | --- |
| **Outcome** | **Exposure** | **β** | **Lower** | **Upper** | **p-value** |
| Global QoL | Intercept | 58.905 | 40.111 | 77.699 | <0.001 |
| Global QoL | Immunotherapy | 0.159 | -9.431 | 9.748 | 0.974 |
| Global QoL | Chemotherapy | -3.538 | -12.268 | 5.193 | 0.426 |
| Global QoL | Radiotherapy | -7.612 | -18.452 | 3.229 | 0.168 |
| Global QoL | Surgery | 1.848 | -4.832 | 8.529 | 0.586 |
| Global QoL | Other therapy | 1.284 | -9.130 | 11.697 | 0.808 |
| **Model 1.2** |  |  | **95% CI** | |  |
| **Outcome** | **Exposure** | **OR** | **Lower** | **Upper** | **p-value** |
| Emotional | Intercept | 1.182 | 0.151 | 9.224 | 0.873 |
| Emotional | Immunotherapy | 1.308 | 0.458 | 3.608 | 0.606 |
| Emotional | Chemotherapy | 1.276 | 0.450 | 3.390 | 0.632 |
| Emotional | Radiotherapy | 0.823 | 0.180 | 3.048 | 0.783 |
| Emotional | Surgery | 1.144 | 0.531 | 2.392 | 0.725 |
| Emotional | Other therapy | 2.276 | 0.718 | 7.206 | 0.157 |
| Social | Intercept | 0.518 | 0.040 | 6.339 | 0.610 |
| Social | Immunotherapy | 2.669 | 0.839 | 7.871 | 0.082 |
| Social | Chemotherapy | 1.075 | 0.275 | 3.429 | 0.908 |
| Social | Radiotherapy | 2.415 | 0.504 | 9.631 | 0.231 |
| Social | Surgery | 0.700 | 0.241 | 1.787 | 0.481 |
| Social | Other therapy | 0.362 | 0.044 | 1.712 | 0.258 |
| Role | Intercept | 2.167 | 0.264 | 18.138 | 0.472 |
| Role | Immunotherapy | 1.244 | 0.421 | 3.524 | 0.684 |
| Role | Chemotherapy | 2.727 | 1.028 | 7.314 | 0.043 |
| Role | Radiotherapy | 3.186 | 0.881 | 11.818 | 0.075 |
| Role | Surgery | 1.314 | 0.620 | 2.750 | 0.470 |
| Role | Other therapy | 0.596 | 0.137 | 2.141 | 0.450 |
| Physical | Intercept | 0.166 | 0.017 | 1.514 | 0.113 |
| Physical | Immunotherapy | 2.185 | 0.661 | 8.636 | 0.223 |
| Physical | Chemotherapy | 2.163 | 0.767 | 6.611 | 0.155 |
| Physical | Radiotherapy | 0.641 | 0.153 | 2.547 | 0.528 |
| Physical | Surgery | 0.913 | 0.419 | 2.011 | 0.820 |
| Physical | Other therapy | 2.281 | 0.566 | 12.135 | 0.278 |
| Cognitive | Intercept | 0.748 | 0.083 | 6.554 | 0.794 |
| Cognitive | Immunotherapy | 1.402 | 0.456 | 4.037 | 0.539 |
| Cognitive | Chemotherapy | 0.875 | 0.256 | 2.564 | 0.817 |
| Cognitive | Radiotherapy | 1.934 | 0.469 | 7.058 | 0.331 |
| Cognitive | Surgery | 1.541 | 0.706 | 3.277 | 0.266 |
| Cognitive | Other therapy | 1.516 | 0.438 | 4.897 | 0.493 |
| Fatigue | Intercept | 2.658 | 0.344 | 21.239 | 0.351 |
| Fatigue | Immunotherapy | 0.892 | 0.303 | 2.536 | 0.831 |
| Fatigue | Chemotherapy | 2.615 | 0.970 | 7.400 | 0.061 |
| Fatigue | Radiotherapy | 2.508 | 0.733 | 9.211 | 0.147 |
| Fatigue | Surgery | 0.929 | 0.443 | 1.921 | 0.844 |
| Fatigue | Other therapy | 2.593 | 0.796 | 9.253 | 0.122 |
| Pain | Intercept | 0.678 | 0.086 | 5.268 | 0.710 |
| Pain | Immunotherapy | 0.810 | 0.262 | 2.277 | 0.699 |
| Pain | Chemotherapy | 1.577 | 0.614 | 3.989 | 0.336 |
| Pain | Radiotherapy | 0.680 | 0.180 | 2.227 | 0.540 |
| Pain | Surgery | 0.887 | 0.413 | 1.833 | 0.751 |
| Pain | Other therapy | 1.596 | 0.526 | 4.743 | 0.398 |
| Nausea/Vomiting | Intercept | 1.201 | 0.147 | 9.876 | 0.864 |
| Nausea/Vomiting | Immunotherapy | 0.506 | 0.153 | 1.470 | 0.231 |
| Nausea/Vomiting | Chemotherapy | 0.660 | 0.219 | 1.797 | 0.433 |
| Nausea/Vomiting | Radiotherapy | 1.712 | 0.488 | 5.738 | 0.385 |
| Nausea/Vomiting | Surgery | 0.695 | 0.317 | 1.457 | 0.347 |
| Nausea/Vomiting | Other therapy | 0.080 | 0.004 | 0.452 | 0.020 |
| Dyspnoea | Intercept | 0.241 | 0.032 | 1.760 | 0.164 |
| Dyspnoea | Immunotherapy | 1.005 | 0.343 | 2.837 | 0.993 |
| Dyspnoea | Chemotherapy | 2.450 | 0.978 | 6.320 | 0.057 |
| Dyspnoea | Radiotherapy | 0.840 | 0.248 | 2.690 | 0.771 |
| Dyspnoea | Surgery | 1.675 | 0.821 | 3.439 | 0.156 |
| Dyspnoea | Other therapy | 1.850 | 0.587 | 5.956 | 0.291 |
| Insomnia | Intercept | 1.861 | 0.163 | 21.141 | 0.615 |
| Insomnia | Immunotherapy | 2.484 | 0.780 | 7.447 | 0.110 |
| Insomnia | Chemotherapy | 2.007 | 0.661 | 5.711 | 0.201 |
| Insomnia | Radiotherapy | 2.373 | 0.605 | 8.376 | 0.190 |
| Insomnia | Surgery | 2.214 | 0.967 | 4.958 | 0.055 |
| Insomnia | Other therapy | 1.208 | 0.238 | 4.678 | 0.798 |
| Loss of appetite | Intercept | 0.037 | 0.002 | 0.643 | 0.029 |
| Loss of appetite | Immunotherapy | 0.848 | 0.196 | 3.004 | 0.809 |
| Loss of appetite | Chemotherapy | 0.591 | 0.113 | 2.169 | 0.473 |
| Loss of appetite | Radiotherapy | 1.120 | 0.180 | 5.021 | 0.891 |
| Loss of appetite | Surgery | 0.398 | 0.116 | 1.125 | 0.107 |
| Loss of appetite | Other therapy | - | - | - | - |
| Constipation | Intercept | 0.009 | 0.000 | 0.483 | 0.027 |
| Constipation | Immunotherapy | 1.458 | 0.228 | 6.976 | 0.658 |
| Constipation | Chemotherapy | 1.955 | 0.407 | 7.859 | 0.365 |
| Constipation | Radiotherapy | 3.228 | 0.416 | 20.266 | 0.225 |
| Constipation | Surgery | 0.952 | 0.212 | 3.425 | 0.944 |
| Constipation | Other therapy | - | - | - | - |
| Diarrhoea | Intercept | 0.205 | 0.022 | 1.734 | 0.151 |
| Diarrhoea | Immunotherapy | 0.200 | 0.030 | 0.783 | 0.044 |
| Diarrhoea | Chemotherapy | 0.418 | 0.124 | 1.184 | 0.124 |
| Diarrhoea | Radiotherapy | 3.386 | 1.073 | 11.365 | 0.039 |
| Diarrhoea | Surgery | 1.041 | 0.487 | 2.161 | 0.915 |
| Diarrhoea | Other therapy | 0.334 | 0.069 | 1.175 | 0.120 |
| Financial difficulties | Intercept | 4.922 | 0.490 | 50.460 | 0.176 |
| Financial difficulties | Immunotherapy | 2.092 | 0.691 | 6.033 | 0.176 |
| Financial difficulties | Chemotherapy | 0.539 | 0.134 | 1.734 | 0.336 |
| Financial difficulties | Radiotherapy | 1.715 | 0.448 | 5.735 | 0.398 |
| Financial difficulties | Surgery | 0.953 | 0.386 | 2.205 | 0.913 |
| Financial difficulties | Other therapy | 2.296 | 0.663 | 7.426 | 0.171 |
| **Model 2.1** |  |  | **95% CI** | |  |
| **Outcome** | **Exposure** | **β** | **Lower** | **Upper** | **p-value** |
| Global QoL | Intercept | 56.021 | 37.458 | 74.583 | <0.001 |
| Global QoL | Heart condition | 3.033 | -4.721 | 10.788 | 0.442 |
| Global QoL | Stroke/CVA | 8.048 | -22.758 | 38.854 | 0.607 |
| Global QoL | High blood pressure | 3.233 | -3.174 | 9.639 | 0.321 |
| Global QoL | Asthma/Chronic bronchitis/COPD | 8.207 | -1.687 | 18.101 | 0.104 |
| Global QoL | Diabetes | -1.601 | -9.288 | 6.087 | 0.682 |
| Global QoL | Ulcer | 1.453 | -20.141 | 23.047 | 0.895 |
| Global QoL | Kidney disease | -2.547 | -18.789 | 13.696 | 0.758 |
| Global QoL | Liver disease | -1.991 | -12.079 | 8.096 | 0.698 |
| Global QoL | Anaemia/Other blood condition | -9.849 | -20.831 | 1.133 | 0.079 |
| Global QoL | Thyroid disease | -3.494 | -14.358 | 7.370 | 0.527 |
| Global QoL | Depression | -19.339 | -37.806 | -0.871 | 0.040 |
| Global QoL | Arthritis | -4.017 | -17.306 | 9.271 | 0.552 |
| Global QoL | Backpain | -11.884 | -18.937 | -4.831 | 0.001 |
| Global QoL | Rheumatism | -6.972 | -21.559 | 7.615 | 0.347 |
| Global QoL | Other comorbidities | -1.626 | -8.087 | 4.835 | 0.621 |
| **Model 2.2** |  |  | **95% CI** | |  |
| **Outcome** | **Exposure** | **OR** | **Lower** | **Upper** | **p-value** |
| Emotional | Intercept | 1.390 | 0.183 | 10.576 | 0.750 |
| Emotional | Heart condition | 0.472 | 0.165 | 1.203 | 0.134 |
| Emotional | Stroke/CVA | - | - | - | - |
| Emotional | High blood pressure | 1.468 | 0.724 | 2.936 | 0.280 |
| Emotional | Asthma/Chronic bronchitis/COPD | 0.567 | 0.143 | 1.838 | 0.374 |
| Emotional | Diabetes | 1.808 | 0.793 | 4.076 | 0.153 |
| Emotional | Ulcer | - | - | - | - |
| Emotional | Kidney disease | 0.493 | 0.049 | 3.557 | 0.505 |
| Emotional | Liver disease | 2.315 | 0.753 | 7.005 | 0.134 |
| Emotional | Anaemia/Other blood condition | 2.901 | 0.893 | 9.689 | 0.076 |
| Emotional | Thyroid disease | 1.340 | 0.393 | 4.326 | 0.626 |
| Emotional | Arthritis | 1.640 | 0.362 | 6.607 | 0.494 |
| Emotional | Backpain | 1.948 | 0.892 | 4.223 | 0.091 |
| Emotional | Rheumatism | 1.128 | 0.193 | 5.532 | 0.884 |
| Emotional | Other comorbidities | 0.950 | 0.448 | 1.948 | 0.891 |
| Social | Intercept | 0.541 | 0.044 | 6.213 | 0.624 |
| Social | Heart condition | 0.401 | 0.093 | 1.311 | 0.167 |
| Social | Stroke/CVA | 8.925 | 0.220 | 381.754 | 0.199 |
| Social | High blood pressure | 0.942 | 0.363 | 2.242 | 0.897 |
| Social | Asthma/Chronic bronchitis/COPD | 1.078 | 0.237 | 3.833 | 0.914 |
| Social | Diabetes | 1.732 | 0.629 | 4.412 | 0.264 |
| Social | Ulcer | - | - | - | - |
| Social | Kidney disease | 0.246 | 0.009 | 2.598 | 0.301 |
| Social | Liver disease | 3.633 | 1.016 | 12.367 | 0.040 |
| Social | Anaemia/Other blood condition | 2.619 | 0.684 | 9.149 | 0.139 |
| Social | Thyroid disease | 1.280 | 0.303 | 4.514 | 0.715 |
| Social | Depression | 0.205 | 0.005 | 2.310 | 0.277 |
| Social | Arthritis | 1.511 | 0.260 | 7.087 | 0.616 |
| Social | Backpain | 2.827 | 1.134 | 6.850 | 0.022 |
| Social | Rheumatism | 4.384 | 0.861 | 22.776 | 0.070 |
| Social | Other comorbidities | 1.035 | 0.404 | 2.423 | 0.939 |
| Role | Intercept | 2.564 | 0.330 | 20.437 | 0.369 |
| Role | Heart condition | 0.802 | 0.308 | 1.977 | 0.640 |
| Role | Stroke/CVA | 1.290 | 0.015 | 57.983 | 0.897 |
| Role | High blood pressure | 1.081 | 0.519 | 2.202 | 0.833 |
| Role | Asthma/Chronic bronchitis/COPD | 0.751 | 0.208 | 2.393 | 0.640 |
| Role | Diabetes | 2.129 | 0.924 | 4.922 | 0.075 |
| Role | Ulcer | - | - | - | - |
| Role | Kidney disease | 0.341 | 0.030 | 2.641 | 0.332 |
| Role | Liver disease | 2.847 | 0.928 | 9.136 | 0.069 |
| Role | Anaemia/Other blood condition | 3.043 | 0.897 | 10.967 | 0.077 |
| Role | Thyroid disease | 2.377 | 0.710 | 8.605 | 0.165 |
| Role | Depression | 7.312 | 0.779 | 193.726 | 0.126 |
| Role | Arthritis | 0.715 | 0.102 | 3.718 | 0.706 |
| Role | Backpain | 3.028 | 1.386 | 6.738 | 0.006 |
| Role | Rheumatism | 3.504 | 0.617 | 24.408 | 0.166 |
| Role | Other comorbidities | 1.453 | 0.706 | 2.965 | 0.305 |
| Physical | Intercept | 0.216 | 0.025 | 1.820 | 0.160 |
| Physical | Heart condition | 1.621 | 0.634 | 4.373 | 0.322 |
| Physical | Stroke/CVA | - | - | - | - |
| Physical | High blood pressure | 1.415 | 0.658 | 3.135 | 0.381 |
| Physical | Asthma/Chronic bronchitis/COPD | 0.639 | 0.186 | 2.243 | 0.473 |
| Physical | Diabetes | 3.304 | 1.195 | 10.816 | 0.030 |
| Physical | Ulcer | 0.182 | 0.006 | 2.684 | 0.240 |
| Physical | Kidney disease | 4.226 | 0.392 | 157.469 | 0.309 |
| Physical | Liver disease | 5.278 | 1.132 | 40.035 | 0.057 |
| Physical | Anaemia/Other blood condition | 3.077 | 0.724 | 21.251 | 0.171 |
| Physical | Thyroid disease | 2.865 | 0.663 | 20.032 | 0.204 |
| Physical | Depression | - | - | - | - |
| Physical | Arthritis | 0.956 | 0.134 | 8.199 | 0.964 |
| Physical | Backpain | 3.588 | 1.456 | 9.999 | 0.009 |
| Physical | Rheumatism | - | - | - | - |
| Physical | Other comorbidities | 2.795 | 1.262 | 6.657 | 0.015 |
| Cognitive | Intercept | 0.849 | 0.098 | 7.209 | 0.881 |
| Cognitive | Heart condition | 0.571 | 0.187 | 1.520 | 0.288 |
| Cognitive | Stroke/CVA | - | - | - | - |
| Cognitive | High blood pressure | 1.075 | 0.495 | 2.249 | 0.850 |
| Cognitive | Asthma/Chronic bronchitis/COPD | 0.536 | 0.109 | 1.935 | 0.382 |
| Cognitive | Diabetes | 1.703 | 0.695 | 4.021 | 0.230 |
| Cognitive | Ulcer | 1.673 | 0.073 | 18.682 | 0.687 |
| Cognitive | Kidney disease | 0.518 | 0.053 | 3.481 | 0.525 |
| Cognitive | Liver disease | 1.745 | 0.509 | 5.503 | 0.351 |
| Cognitive | Anaemia/Other blood condition | 2.208 | 0.615 | 7.630 | 0.210 |
| Cognitive | Thyroid disease | 2.223 | 0.676 | 7.310 | 0.181 |
| Cognitive | Depression | - | - | - | - |
| Cognitive | Arthritis | 1.901 | 0.332 | 8.804 | 0.428 |
| Cognitive | Backpain | 2.401 | 1.068 | 5.330 | 0.032 |
| Cognitive | Rheumatism | 1.465 | 0.251 | 7.366 | 0.647 |
| Cognitive | Other comorbidities | 0.811 | 0.356 | 1.737 | 0.601 |
| Fatigue | Intercept | 3.640 | 0.495 | 27.970 | 0.208 |
| Fatigue | Heart condition | 0.765 | 0.317 | 1.790 | 0.541 |
| Fatigue | Stroke/CVA | - | - | - | - |
| Fatigue | High blood pressure | 0.799 | 0.395 | 1.590 | 0.525 |
| Fatigue | Asthma/Chronic bronchitis/COPD | 0.268 | 0.059 | 0.928 | 0.055 |
| Fatigue | Diabetes | 0.924 | 0.390 | 2.139 | 0.854 |
| Fatigue | Ulcer | 1.577 | 0.143 | 21.582 | 0.706 |
| Fatigue | Kidney disease | 0.526 | 0.067 | 3.413 | 0.512 |
| Fatigue | Liver disease | 2.364 | 0.775 | 7.876 | 0.139 |
| Fatigue | Anaemia/Other blood condition | 4.555 | 1.251 | 20.033 | 0.029 |
| Fatigue | Thyroid disease | 2.895 | 0.810 | 13.730 | 0.128 |
| Fatigue | Depression | - | - | - | - |
| Fatigue | Arthritis | 0.784 | 0.133 | 3.810 | 0.769 |
| Fatigue | Backpain | 2.201 | 1.029 | 4.831 | 0.044 |
| Fatigue | Rheumatism | 5.389 | 1.008 | 44.546 | 0.069 |
| Fatigue | Other comorbidities | 1.563 | 0.776 | 3.189 | 0.213 |
| Pain | Intercept | 0.801 | 0.106 | 5.988 | 0.829 |
| Pain | Heart condition | 0.640 | 0.253 | 1.513 | 0.324 |
| Pain | Stroke/CVA | 0.658 | 0.015 | 22.128 | 0.803 |
| Pain | High blood pressure | 1.057 | 0.517 | 2.107 | 0.877 |
| Pain | Asthma/Chronic bronchitis/COPD | 1.782 | 0.606 | 5.178 | 0.284 |
| Pain | Diabetes | 0.832 | 0.342 | 1.917 | 0.674 |
| Pain | Ulcer | 0.651 | 0.029 | 6.416 | 0.732 |
| Pain | Kidney disease | 1.543 | 0.253 | 8.624 | 0.619 |
| Pain | Liver disease | 0.992 | 0.313 | 2.935 | 0.989 |
| Pain | Anaemia/Other blood condition | 5.922 | 1.800 | 23.403 | 0.005 |
| Pain | Thyroid disease | 1.700 | 0.511 | 5.601 | 0.377 |
| Pain | Depression | 3.846 | 0.539 | 36.615 | 0.191 |
| Pain | Arthritis | 0.825 | 0.169 | 3.382 | 0.797 |
| Pain | Rheumatism | 2.326 | 0.476 | 12.003 | 0.292 |
| Pain | Other comorbidities | 1.368 | 0.684 | 2.693 | 0.368 |
| Nausea/Vomiting | Intercept | 1.019 | 0.134 | 7.725 | 0.985 |
| Nausea/Vomiting | Heart condition | 0.775 | 0.298 | 1.872 | 0.584 |
| Nausea/Vomiting | Stroke/CVA | - | - | - | - |
| Nausea/Vomiting | High blood pressure | 0.988 | 0.479 | 1.984 | 0.974 |
| Nausea/Vomiting | Asthma/Chronic bronchitis/COPD | 0.924 | 0.275 | 2.766 | 0.891 |
| Nausea/Vomiting | Diabetes | 0.992 | 0.409 | 2.283 | 0.985 |
| Nausea/Vomiting | Ulcer | 0.911 | 0.040 | 9.552 | 0.941 |
| Nausea/Vomiting | Kidney disease | 0.617 | 0.074 | 3.573 | 0.610 |
| Nausea/Vomiting | Liver disease | 1.816 | 0.602 | 5.421 | 0.281 |
| Nausea/Vomiting | Anaemia/Other blood condition | 2.901 | 0.912 | 9.828 | 0.074 |
| Nausea/Vomiting | Thyroid disease | 1.878 | 0.597 | 6.179 | 0.282 |
| Nausea/Vomiting | Depression | 6.397 | 0.749 | 143.419 | 0.130 |
| Nausea/Vomiting | Arthritis | 1.073 | 0.195 | 4.709 | 0.929 |
| Nausea/Vomiting | Backpain | 0.938 | 0.408 | 2.049 | 0.875 |
| Nausea/Vomiting | Rheumatism | 1.303 | 0.228 | 6.516 | 0.749 |
| Nausea/Vomiting | Other comorbidities | 1.507 | 0.745 | 3.012 | 0.248 |
| Dyspnoea | Intercept | 0.356 | 0.050 | 2.482 | 0.300 |
| Dyspnoea | Heart condition | 0.739 | 0.319 | 1.661 | 0.469 |
| Dyspnoea | Stroke/CVA | - | - | - | - |
| Dyspnoea | High blood pressure | 0.813 | 0.406 | 1.597 | 0.551 |
| Dyspnoea | Diabetes | 1.060 | 0.460 | 2.406 | 0.889 |
| Dyspnoea | Ulcer | 1.028 | 0.076 | 10.943 | 0.981 |
| Dyspnoea | Kidney disease | 2.903 | 0.490 | 25.961 | 0.269 |
| Dyspnoea | Liver disease | 2.717 | 0.912 | 8.750 | 0.078 |
| Dyspnoea | Anaemia/Other blood condition | 3.958 | 1.183 | 15.789 | 0.033 |
| Dyspnoea | Thyroid disease | 1.521 | 0.443 | 5.343 | 0.502 |
| Dyspnoea | Depression | 6.492 | 0.671 | 166.755 | 0.154 |
| Dyspnoea | Arthritis | 0.674 | 0.125 | 2.965 | 0.614 |
| Dyspnoea | Backpain | 1.285 | 0.600 | 2.739 | 0.514 |
| Dyspnoea | Rheumatism | 3.875 | 0.635 | 36.748 | 0.174 |
| Dyspnoea | Other comorbidities | 0.623 | 0.307 | 1.225 | 0.178 |
| Insomnia | Intercept | 2.760 | 0.255 | 30.194 | 0.402 |
| Insomnia | Heart condition | 0.980 | 0.321 | 2.667 | 0.970 |
| Insomnia | Stroke/CVA | 5.584 | 0.130 | 197.135 | 0.310 |
| Insomnia | High blood pressure | 0.888 | 0.358 | 2.046 | 0.788 |
| Insomnia | Asthma/Chronic bronchitis/COPD | 3.854 | 1.230 | 11.923 | 0.018 |
| Insomnia | Diabetes | 1.349 | 0.496 | 3.408 | 0.538 |
| Insomnia | Ulcer | 1.791 | 0.076 | 19.295 | 0.650 |
| Insomnia | Kidney disease | 1.307 | 0.149 | 8.519 | 0.787 |
| Insomnia | Liver disease | 1.418 | 0.377 | 4.651 | 0.580 |
| Insomnia | Anaemia/Other blood condition | 4.821 | 1.428 | 16.901 | 0.011 |
| Insomnia | Thyroid disease | 1.254 | 0.297 | 4.421 | 0.737 |
| Insomnia | Depression | 2.698 | 0.330 | 22.309 | 0.337 |
| Insomnia | Arthritis | 0.714 | 0.090 | 3.896 | 0.719 |
| Insomnia | Backpain | 1.150 | 0.430 | 2.813 | 0.769 |
| Insomnia | Rheumatism | 3.510 | 0.581 | 20.606 | 0.156 |
| Insomnia | Other comorbidities | 0.793 | 0.313 | 1.835 | 0.605 |
| Loss of appetite | Intercept | 0.053 | 0.003 | 0.764 | 0.036 |
| Loss of appetite | Heart condition | 0.808 | 0.229 | 2.435 | 0.720 |
| Loss of appetite | Stroke/CVA | - | - | - | - |
| Loss of appetite | High blood pressure | 1.756 | 0.733 | 4.038 | 0.193 |
| Loss of appetite | Asthma/Chronic bronchitis/COPD | 1.395 | 0.282 | 5.422 | 0.653 |
| Loss of appetite | Diabetes | 0.538 | 0.143 | 1.641 | 0.311 |
| Loss of appetite | Ulcer | - | - | - | - |
| Loss of appetite | Kidney disease | 1.367 | 0.136 | 9.861 | 0.766 |
| Loss of appetite | Liver disease | 5.028 | 1.530 | 16.371 | 0.007 |
| Loss of appetite | Anaemia/Other blood condition | 0.821 | 0.134 | 3.649 | 0.810 |
| Loss of appetite | Thyroid disease | 2.143 | 0.508 | 7.821 | 0.264 |
| Loss of appetite | Depression | 7.632 | 0.837 | 88.663 | 0.078 |
| Loss of appetite | Arthritis | 0.847 | 0.119 | 4.407 | 0.854 |
| Loss of appetite | Backpain | 0.882 | 0.279 | 2.434 | 0.819 |
| Loss of appetite | Rheumatism | 5.181 | 0.940 | 30.706 | 0.056 |
| Loss of appetite | Other comorbidities | 0.310 | 0.079 | 0.928 | 0.058 |
| Constipation | Intercept | 0.008 | 0.000 | 0.343 | 0.016 |
| Constipation | Heart condition | 0.519 | 0.058 | 2.723 | 0.487 |
| Constipation | Stroke/CVA | - | - | - | - |
| Constipation | High blood pressure | 0.293 | 0.039 | 1.294 | 0.156 |
| Constipation | Asthma/Chronic bronchitis/COPD | 4.052 | 0.776 | 18.225 | 0.077 |
| Constipation | Diabetes | 1.056 | 0.198 | 4.211 | 0.943 |
| Constipation | Ulcer | - | - | - | - |
| Constipation | Kidney disease | 2.094 | 0.081 | 24.393 | 0.586 |
| Constipation | Liver disease | 2.027 | 0.316 | 9.711 | 0.407 |
| Constipation | Anaemia/Other blood condition | 6.568 | 1.515 | 27.945 | 0.010 |
| Constipation | Thyroid disease | 1.613 | 0.195 | 8.751 | 0.608 |
| Constipation | Depression | 8.408 | 0.790 | 111.742 | 0.079 |
| Constipation | Arthritis | 1.411 | 0.096 | 10.741 | 0.765 |
| Constipation | Backpain | 0.955 | 0.189 | 3.721 | 0.950 |
| Constipation | Rheumatism | 1.284 | 0.048 | 12.485 | 0.850 |
| Constipation | Other comorbidities | 0.985 | 0.230 | 3.418 | 0.982 |
| Diarrhoea | Intercept | 0.216 | 0.027 | 1.653 | 0.145 |
| Diarrhoea | Heart condition | 1.031 | 0.419 | 2.419 | 0.945 |
| Diarrhoea | Stroke/CVA | - | - | - | - |
| Diarrhoea | High blood pressure | 0.546 | 0.244 | 1.142 | 0.121 |
| Diarrhoea | Asthma/Chronic bronchitis/COPD | 1.024 | 0.314 | 3.032 | 0.966 |
| Diarrhoea | Diabetes | 1.000 | 0.407 | 2.313 | 1.000 |
| Diarrhoea | Ulcer | 0.577 | 0.025 | 5.717 | 0.663 |
| Diarrhoea | Kidney disease | 1.117 | 0.176 | 6.242 | 0.901 |
| Diarrhoea | Liver disease | 1.567 | 0.523 | 4.512 | 0.407 |
| Diarrhoea | Anaemia/Other blood condition | 0.816 | 0.201 | 2.748 | 0.756 |
| Diarrhoea | Thyroid disease | 1.140 | 0.324 | 3.626 | 0.829 |
| Diarrhoea | Depression | 3.144 | 0.421 | 28.865 | 0.265 |
| Diarrhoea | Arthritis | 0.845 | 0.165 | 3.604 | 0.826 |
| Diarrhoea | Backpain | 1.480 | 0.694 | 3.088 | 0.300 |
| Diarrhoea | Rheumatism | 1.680 | 0.283 | 9.385 | 0.546 |
| Diarrhoea | Other comorbidities | 1.313 | 0.647 | 2.608 | 0.441 |
| Financial difficulties | Intercept | 3.424 | 0.364 | 32.662 | 0.281 |
| Financial difficulties | Heart condition | 0.813 | 0.296 | 2.043 | 0.672 |
| Financial difficulties | Stroke/CVA | 3.912 | 0.090 | 136.567 | 0.420 |
| Financial difficulties | High blood pressure | 1.731 | 0.803 | 3.641 | 0.152 |
| Financial difficulties | Asthma/Chronic bronchitis/COPD | 2.151 | 0.698 | 6.311 | 0.167 |
| Financial difficulties | Diabetes | 1.854 | 0.780 | 4.253 | 0.151 |
| Financial difficulties | Ulcer | 4.157 | 0.339 | 46.367 | 0.229 |
| Financial difficulties | Kidney disease | 0.779 | 0.105 | 4.590 | 0.790 |
| Financial difficulties | Liver disease | 2.519 | 0.804 | 7.622 | 0.103 |
| Financial difficulties | Anaemia/Other blood condition | 1.831 | 0.493 | 6.197 | 0.340 |
| Financial difficulties | Thyroid disease | 0.265 | 0.014 | 1.493 | 0.219 |
| Financial difficulties | Depression | 2.576 | 0.343 | 20.224 | 0.344 |
| Financial difficulties | Arthritis | 1.087 | 0.203 | 4.795 | 0.915 |
| Financial difficulties | Backpain | 1.280 | 0.533 | 2.910 | 0.565 |
| Financial difficulties | Rheumatism | 1.021 | 0.148 | 5.645 | 0.982 |
| Financial difficulties | Other comorbidities | 1.109 | 0.494 | 2.370 | 0.795 |
| -“: very large point estimates (>100) with wide confidence interval of length > 200 | | | | | |
